# Role of Toll-like receptor 2 during infection of *Leptospira* spp: A systematic review

**DOI:** 10.1371/journal.pone.0312466

**Published:** 2024-12-27

**Authors:** Chamila Kappagoda, Indika Senavirathna, Thilini Agampodi, Suneth Buddhika Agampodi

**Affiliations:** 1 Department of Community Medicine, Faculty of Medicine and Allied Sciences, Rajarata University of Sri Lanka, Saliyapura, Anuradhapura, Sri Lanka; 2 Department of Biochemistry, Faculty of Medicine and Allied Sciences, Rajarata University of Sri Lanka, Saliyapura, Anuradhapura, Sri Lanka; 3 International Vaccine Institute, Seoul, Republic of Korea; 4 Department of Internal Medicine, Section of Infectious Diseases, School of Medicine, Yale University, New Haven, Connecticut, United States of America; Universita degli Studi di Parma, ITALY

## Abstract

The involvement of Toll-like receptor 2 (TLR2) in leptospirosis is poorly understood. Our systematic review examined its role across *in-vitro*, *in-vivo*, *ex-vivo*, and human studies. Original articles published in English up to January 2024, exploring the role of TLR2 during leptospirosis, were selected from databases including PubMed, Web of Science, Scopus, Trip, and Google Scholar. Cochrane guidelines and Preferred Reporting Items for Systematic Reviews and Meta-Analyses were followed by this systematic review. The National Institute of Health Quality Assessment tool, Systematic Review Centre for Laboratory Animal Experimentation risk of bias tool, and Office of Health Assessment and Translation extended tool were used to assess the risk of bias of the studies. Out of 2458 studies retrieved, 35 were selected for the systematic review. These comprised 3 human, 17 *in-vitro*, 5 *in-vivo*, 3 *ex-vivo*, and 7 studies with combined experimental models. We assessed the direct TLR2 expression and indirect TLR2 involvement via the secretion/mRNA expression of immune effectors during leptospirosis. Notably, we observed the secretion/mRNA expression of several cytokines (IL6, IL8, IL-1β, TNFα, IFNγ, IL10, CCL2/MCP-1, CCL10, COX2, CXCL1/KC, CXCL2/MIP2) and immune effectors (hBD2, iNOS, Fibronectin, Oxygen, and Nitrogen reactive species) as key aspects of host TLR2 responses during leptospirosis. Even though increased TLR2 expression in *in-vivo* and *in-vitro* studies was evident, human studies reported mixed results showing that the postulated effect of TLR2 response based on other studies may not be valid for human leptospirosis. Besides the role of TLR2 in response to leptospirosis, the involvement of TLR4 and TLR5 was identified in *in-vitro* and *in-vivo* studies. TLR2 expression is inconclusive during human leptospirosis and further studies are needed to examine the immune effector regulation, through TLR2 for mitigating the harmful effects and promoting effective immune responses.

## Introduction

Leptospirosis is a globally widespread, infectious zoonosis caused by a spiral-shaped bacterium belonging to the genus *Leptospira* [1, 2]. Pathogenic *Leptospira* spp. play a significant role in infecting humans, resulting in a wide range of clinical symptoms ranging from febrile illness to multi-organ failures [3]. Varied immune responses among hosts play a crucial role in influencing disease progression alongside pathogenesis and epidemiological factors of the infectious organism. Although a variety of host Pattern Recognition Receptors (PRRs) are possessed by innate immune cells during an infection, Toll-like receptors (TLRs) are among the most studied PRRs in primary infectious disease research [4].

The ‘Toll’ gene was first isolated from *Drosophila melanogaster* (Common fruit fly) by Carl Hashimoto and colleagues in 1988 [5]. The first TLR identified in mammalian tissues was TLR4. Previously named hToll, the gene expression of TLR4 has been reported in monocytes, macrophages, dendritic cells, γδ T cells, and small intestinal cell lines of humans and mice [6]. Since TLR4 was identified in mammals, 13 TLRs (TLR1-TLR13) have been discovered and described in the literature [7]. TLR2 interacts with different types of Pathogen Associated Molecular Patterns (PAMPs) from various pathogens including viruses, fungi, bacteria, parasites which are essential for their survival and virulence [8]. Usually, TLR2 forms heterodimers together with TLR1 or TLR6 on the cell surfaces and also it forms TLR2 homodimers [9] Loading of PAMPs to TLR2 initiates the cascade of reactions to trigger the gene transcription and corresponding cytokine production to clear the pathogens.

Lipopolysaccharides (LPS) and proteins are the major PAMPs of pathogenic *Leptospira* spp. [10–13]. Numerous *in-vitro* and mice model studies have been conducted to study the immune responses against *Leptospira* spp. TLR1, TLR2, TLR4, and TLR6 are considered to have direct involvement in the immune response against *Leptospira* spp. [12, 14–19]. Mice are reservoir hosts for pathogenic *Leptospira* spp. and their TLR4 can recognize LPS of *Leptospira spp*. [12]. Since human TLR4 is unable to identify the LPS of *Leptospira* spp. [20], further exploration is needed regarding the role of TLR2 during leptospirosis. Among the TLRs examined, TLR2 has been proposed to play a key role in recognizing lipoproteins in pathogens. This hypothesis is supported by the observation that TLR2-deficient mice exhibit increased susceptibility to the gram-positive bacterium *Streptococcus pneumonia* compared to wild-type mice [21]. Atomic force microscope studies have provided direct evidence of TLR2 interaction with LipL32 expressed on the *Leptospira* cell surface [22]. Interaction of pathogenic *Leptospira* spp. with human PRRs initiates a cascade of reactions to generate the responses via secretion of immune effectors; antimicrobial peptides, cytokines, and chemokine that attract leukocytes to the infection site to destroy the pathogens [13, 14].

The response of the TLR2 was studied majorly in *in-vitro*, *in-vivo*, *ex-vivo* models, and scarcely in humans. Though the experimental findings have shown the protective role of TLR2, there has not been a systematic evidence synthesis to shed light on the grey areas of this process. In this paper, we systematically reviewed the published literature on the role of TLR2 during *Leptospira* infection in *in-vitro*, *in-vivo*, and *ex-vivo* experimental models and human studies.

## Methods

The systematic review was carried out per the Cochrane guidelines [23] and reported according to the Preferred Reporting Items for Systematic Reviews and Meta-Analyses Statement 2020 (PRISMA2020) [24]. S3 and S4 Tables show PRISMA Check Lists.

### Eligibility criteria

We included all human, *in-vivo*, *ex-vivo*, and *in-vitro* experimental studies that examined the response of TLR2 during *Leptospira* spp. infection. The key terms were defined based on the PICO (Participants, Intervention, Comparators, and Outcomes) approach. We selected original articles published in English up to January 2024. We included all studies reporting either the infection or inoculation of *Leptospira* spp. Healthy individuals, healthy animals, and uninfected cell lines were the comparators used. We excluded papers that were editorials, book chapters, or responses to authors.

### Information sources

We conducted the search using electronic databases, including PubMed, Web of Science, Scopus, Trip, and Google Scholar, to find relevant articles. Our search strings included medical subject headings (MeSH terms), and keywords. Parenthesis was generated using the terms representing “Toll-like receptor 2” and “Leptospirosis”. We used combined specific search strings modified for each database to identify eligible studies. In the meantime, we manually checked the reference lists of included articles, reviews, and systematic reviews to find additional relevant articles. S1 Table shows the search strings modified for the literature search.

### Selection process

**Fig 1:**
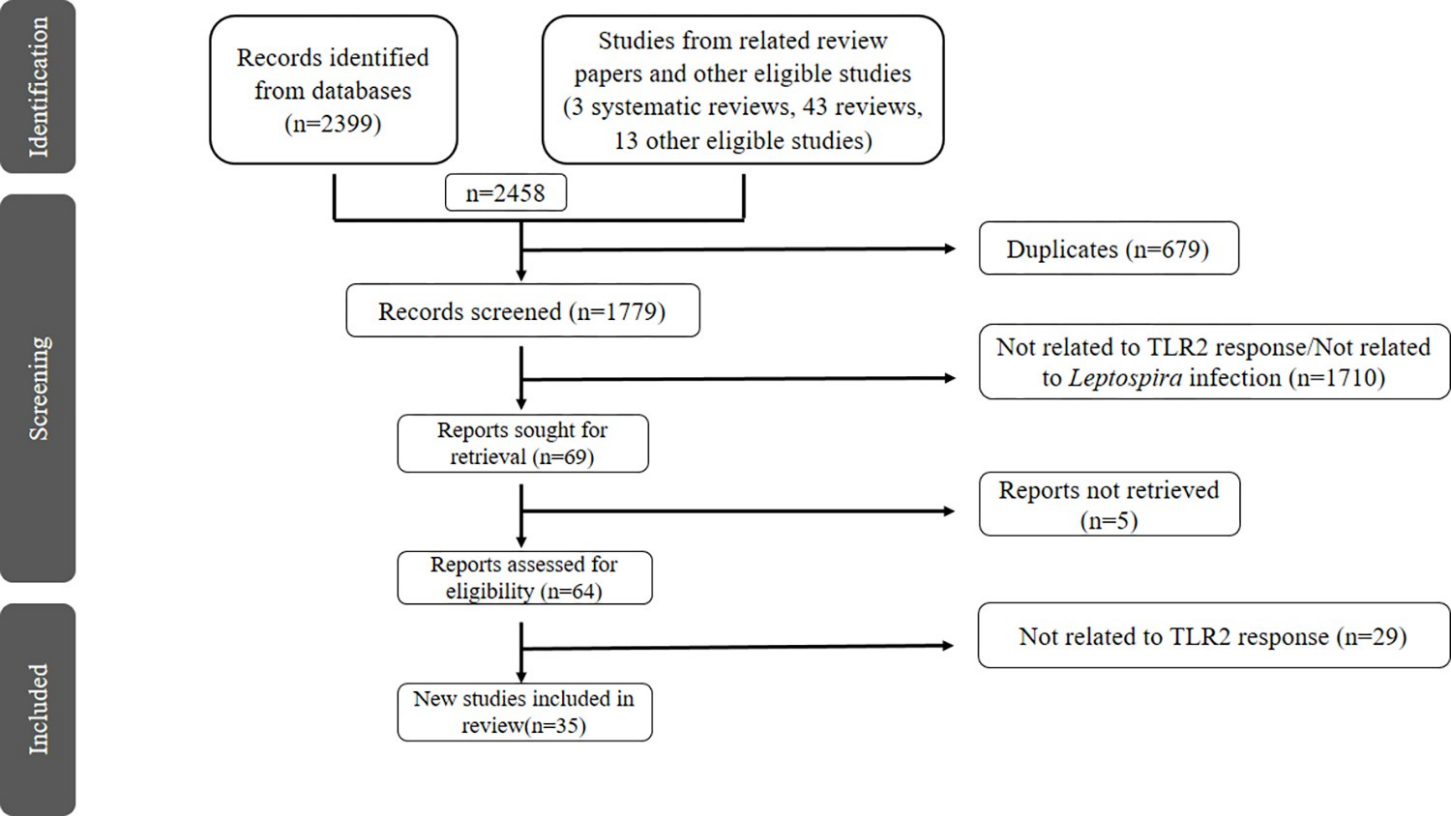
PRISMA flow chart shows study selection for the systematic review.

CK conducted the literature search. The search results were entered into Mendeley software (Mendeley Desktop version 1.19.8). Duplicate studies were screened and removed by the ’check for duplicates’ tool in Mendeley. Title and abstract screening were carried out independently by CK and IS, according to predefined eligibility criteria. The initial screening methodology was performed in duplicate. In case of discrepancies during the study selection, SA served to create a consensus and were resolved by discussions among CK, IS and SA. The full-text screening was conducted independently and in duplicate by CK and IS for the final inclusion of studies. The study selection process was done, adhering to the guidelines outlined in the Preferred Reporting Items for Systematic Reviews and Meta-Analyses (PRISMA) flow chart (Fig 1).

### Data extraction

Data extraction was performed independently and in duplicate in a structured Excel sheet by CK and IS. The extracted data items included Article Citation, Country of origin, Aim/objectives of the study, Study participants (Human/*In-vivo*/*in-vitro*/*ex-vivo* study), Human study (age, *Leptospira* incubation period, diagnosed method, healthy control group, type of the clinical specimen/tissue investigated, experimental method used to investigate the TLR2 response, main finding of TLR2 response), *In-vivo* study (Animal model, infection duration, experimental details that deployed to analyze the TLR2 response, main finding of TLR2 response), *In-vitro*/*Ex-vivo* (Type of the cell line, infection duration, experimental details that deployed to analyze the TLR2 response, main finding of TLR2 response).

### Study risk of bias assessment

The risk of bias in selected studies was assessed independently by CK and IS using appropriate quality assessment tools. Given that the systematic review included human studies, *in-vivo* studies, *in-vitro*, and *ex-vivo* studies, different quality assessment tools were used to assess the risk of bias for each type of study. Specifically, the National Institute of Health Quality Assessment tool- Quality assessment of case-control studies was used for human studies [25], the SYstematic Review Centre for Laboratory animal Experimentation (SYRCLE’s) risk of bias tool was used for *in-vivo* studies [26], and the OHAT (Office of Health Assessment and Translation) extended tool [27] was used for *in-vitro* and *ex-vivo* studies. The risk of bias information was checked and recorded for each study in a table to facilitate data analysis.

### Synthesis methods

Since the data for this study are presented as gene expression profile, cell receptor expression, and immune responses via TLR2 recognition, the data were not suitable for combining quantitatively. A narrative synthesis was conducted with the information reported in the text and tables to summarize and explain the characteristics and findings of the included studies. We explored the relationship and findings both within and between the included studies. Eventually, the role of TLR2 during the infection of *Leptospira* spp. was summarized in the present systematic review.

### Reporting bias assessment

The RoB tool SYRCLE’s presented by SYstematic Review Centre for Laboratory animal Experimentation based on the Cochrane Collaboration Risk of bias (RoB) tool [26] was used to assess the experimental *in-vivo* intervention studies. The checklist (10 questions) evaluates the risk of bias in the studies and addresses selection, performance, attrition, detection, and reporting biases. For *in-vivo* studies, the risk of bias was assessed and reported via the checklist of SYRCLE’s RoB tool. OHAT RoB tool was used to assess the reporting bias in *in-vitro* studies with 11 questions grouped into 6 types of biases (selection, confounding, performance, attrition/exclusion, detection, and selective reporting).

### Certainty assessment

GRADE (Grading of Recommendations Assessment, Development, and Evaluation) [28] domains (imprecision, inconsistency, risk of bias, indirectness, and other) were used to assess the certainty of each important outcome and an overall judgment of whether the evidence supporting a result is of high, moderate, low, or very low certainty.

## Results

### Study selection

A total of 2458 studies were retrieved from an electronic database search and the reference lists of all reviews and other eligible studies. We removed 679 duplicate articles and 1710 articles lacking relevance to the study. An additional full text of 5 studies were not retrieved and 29 were excluded due to outcome differences. Eventually, 35 studies met the eligibility criteria and were included in the systematic review (Fig 1).

### General characteristics of the included studies

The 35 articles selected for this review were published between 2001 and 2023, with 7 articles from China [17–19, 29–32], 7 from Taiwan [33–39], 6 from France [12, 40–44], 3 from the United States [16, 20, 45], 2 from Thailand [46, 47], 3 from India [14, 48, 49], 2 from Brazil [50, 51], 2 from the Netherlands [15, 52], 2 from Japan [53, 54], and 1 from Argentina [55]. The studies encompassed a range of experimental models, including 3 human [41, 42, 51], 5 *in-vivo* [18, 31, 37, 38, 44], 17 *in-vitro* [14, 15, 29, 30, 32–36, 39, 40, 46, 49, 50, 52–54], and 3 *ex-vivo* studies [45, 47, 55]. Additionally, four studies [12, 16, 19, 20] combined *in-vivo* and *in-vitro* models, while two studies [17, 56] used a combination of human, *in-vitro*, and *in-vivo* models. Finally, one study [43] used both *in-vivo* and *ex-vivo* models. All *in-vivo*, *in-vitro*, and *ex-vivo* studies are experimental design studies while human studies are observational studies. S2 Table and S1 Appendix show the General characteristics of the studies.

### TLR2 response in i*n-vitro* and *ex-vivo* studies

#### Direct response

Regardless of the type of non-human cell line and the *Leptospira* stimulant, the stimulated cell lines had an increased TLR2 gene expression compared to unstimulated cell lines [29, 30, 32, 39, 45, 53, 54]. Increased TLR2 gene expression was observed in mouse/rat proximal tubule cells [29, 39], pig embryonic cells [53, 54], bovine cell line [30, 32], and canine whole blood [45]. In terms of stimulants, pathogenic *Leptospira* outer membrane proteins, (LipL32) [39], Loa22 [29], lipopolysaccharide (LPS) [30, 32, 53, 54], *L*. *interrogans* Copenhageni [45] stimulated the cells that were increased TLR2 gene expression. Significantly activated canine, mouse, and human TLR2-expressing reporter cells were observed in a dose-dependent manner [52] but not reported increase of TLR2 gene expression.

Nevertheless, *in-vitro* [33, 35, 36, 39, 49] and *ex-vivo* [47, 55] studies, that used human cell lines as HEK 293 [35, 36, 39, 49], HK2 [33], THP- 1 [49], human oral tissues [47], and human blood [55], did not examine the direct TLR2 response. In contrast with the increased TLR2 gene expression in non-human *in-vitro*/*ex-vivo* studies, TLR2 gene expression in human cells (HK 2) infected with *L*. *interrogans* Autumnalis did not differ from unstimulated cells [46]. However, increased TLR2 expression was observed in the lung tissues of patients who died because of leptospirosis [50]. The *in-vitro* studies observed a TLR2 gene expression within 48 hours of infection, showing the response in the early stage of the disease.

#### Indirect response

Blocked TLR2 / TLR2 deficient cell lines that were stimulated using pathogenic *Leptospira* organism/cell components showed inhibition or decreased cytokine/immune mediator level compared to intact TLR2 cell lines. Even with the heterogeneous stimulants and the cell lines, IL6 [15, 18, 19, 30, 32, 36, 45, 46, 49, 57, 58], IL8 [20, 36, 46, 47, 49], IL 1β [14, 17, 46, 47, 56], TNFα [14, 15, 16, 17, 20, 32, 36, 46, 47, 49, 56], IFNγ [14], IL10 [19, 56], iNOS [29, 39], CCL2/MCP-1 [14, 29, 34–36, 39], hBD2 [46, 47], CCL10 [14, 34–36], COX2 [14], CXCL1/KC [34], NFκB [56], Fibronectin [33] and CXCL2/MIP2 [35] showed the inhibition or decreased level in TLR2 blocked/deficient cell lines. The data convey the involvement of TLR2 in triggering the immune response during the *Leptospira* infection Accumulation of pleiotropic protein P62 increased through TLR2 and TLR4, that mediating cell stress and cell death [40]. Tables 1 and 2 show the characteristics of *in-vitro* and *ex-vivo* studies, respectively.

**Table 1:** *In-vitro* study characteristics.

ID	Cell line/Tissue	Stimulant and incubation period	Method	Main finding	
				Direct Response	In-direct Response
(Goris,2011) [15]	Whole blood (from healthy donors)	*L*. *interrogans* serovar Bataviae strain kariadi-satu. and Lai type Langkawi (6 hrs)	ELISA (TNFα) Cytometric beads array(IL6) (Cell supernatants stored at -70°C)	NA	Inhibition of TNFα production by either TLR2 (250 to 120pg/mL) or TLR4 (250 to 110pg/mL) blockage, not by combination. Blockage of TLR5 significantly reduces the TNFα production (from 250 to 150 pg/mL). Anti TLR2 is moderately effective in decreasing IL6 (from 1700 to 1250pg/mL) production. Combined blocking of TLR2 and TLR4 effectively decreases IL6 (from 1700 to 750pg/mL).
(Yang,2006) [39]	Mouse Proximal tubule cells (PKSV-PR) Human embryonic kidney cells (HEK 293 cells)	*L*. *santarosai* Shermani OMPs—Lipoproteins (48 hrs)	Flowcytometry RT qPCR ELISA	Increased TLR2 expression by 28%, compared to untreated cells, while TLR4 expression was not induced. TLR2 mRNA expression showed a 1.5–1.9 fold increase compared to untreated cells. TLR2 expression increased 2.7-fold for LipL32. 50% reduced TLR2 expression by blockingLipL32. Results indicate that LipL32 can directly affect TLR2 gene expression in renal proximal tubule cells.	As a function of time, TLR2 mRNA was activated within 2 h (24hrs) of stimulation, followed by the increase of Induced CCL2/MCP-1 mRNA expression by 3.1- to 3.4-fold in HEK 293. CCL2/MCP-1 mRNA and a progressive increase of CCL2/MCP-1 secreted measured by ELISA.
(Faisal,2016) [14]	Mouse macrophages (RAW264.7)	Recombinant protein Lsa21 (2 μ g/ml) (24 hrs)	ELISA	NA	Significantly reduced or downregulated (IL6-1700-1100, TNF α-500-400 pg/mL) in TLR2−/− cells stimulated with Lsa21. It was completely abrogated in double- knockout cell lines (TLR2, TLR4−/−).
		(4hrs,24hrs,48hrs)	q RT PCR	NA	Genes (CCL2, CCL10, COX2, IL-1β, IL-6, TNF-α, MCP-1, IFN-γ, iNOS) were significantly reduced or down regulated in TLR2−/− and TLR4−/− cell stimulated with Lsa21 compared with WT cells.
(Guo,2015)-1 [53]	Pig embryonic fibroblast cell line	LPS of *L*. *interrogans* serovar Hebdomadis (6,12 and 24 hrs)	q RT PCR Western blot	Expression of TLR2 mRNA was gradually and monotonically increased (more than 1.5 P<0.05) compared with unstimulated cells. TLR2 protein in cells stimulated by L-LPS was about two-fold that in unstimulated cells.	The pig fibroblast can induce IL-6 and IL-8 within 6 h after being stimulated by L-LPS.
(Guo,2015)-2 [54]	Pig embryonic fibroblast cell line	LPS of *L*. *interrogans* serovar Hebdomadis (3hrs)	q RT PCR Western blot	Expression of TLR2 mRNA was markedly up-regulated to 2.3 folds at 3 h and then rapidly declined at 12 h. than unstimulated cells. TLR2 protein with a molecular mass of 74 kDa was stably expressed at a low level, and the expression level of TLR2 protein was constant until 72 h after stimulation.	Increased IL-6 and IL-8 mRNA expression were caused. The secretion levels of IL-6(220pg/mL) and IL8 (390pg/mL) were induced within 3h.
(Guo, 2016) [32]	Bovine cell line	LPS of *L*. *interrogans* serovar Hardjo (6hrs)	q RT PCR ELISA	The level of TLR2 mRNA expression to that of unstimulated cells at each time point after the start of the incubation was greater than 1.5.	Anti-TLR2 antibody showed a significant inhibitory effect on bovine IL-6 (750-200pg/mL) and TNF-α (200-75pg/mL) production in L-LPS stimulated cells.
(Hsu, 2021) [36]	HEK293 HEK293-TLR2	LipL32 and Loa22 WT rLoa22-LPGN complex	ELISA q RT PCR	NA	rLoa22-LPGN complexes significantly increased mRNA and protein expression levels of CXCL8/IL8 (1–12.5) (1–5), hCCL2/MCP-1(1–9) (1.5–5), and hTNF-α (1–12) (1–4.5) as compared to that of Loa22WT. Results indicated that rLoa22-LPGN stimulated the highest levels of cytokines expression mainly through TLR2.
(Hung,2006)-1 [35]	HEK293 cells	*Leptospira* membrane protein (LMPS)(24hrs) *L*. *santarosai* Shermani	ELISA	NA	Strongly indicate that the action of LMPS on the phosphorylation of p38 and induction of chemokine (CCL2/MCP-1(4200 -1200pg/mL)) and (CXCL2/MIP-2(1500 -500pg/mL)) release requires a functional TLR2.
(Hung,2006)-2 [34]	Murine proximal tubule cells(PTCs)	*Leptospira* membrane lipoprotein (*L*. *santorasai* Shermani) (LMLP) (24hrs)	ELISA	NA	In comparison with control (Control siRNA), TLR2 siRNA, MyD88 siRNA, and TRAF6 siRNA significantly hindered the secretion of CXCL1/KC(2300-1250pg/mL) in murine PTCs.
			q RT PCR	NA	CXCL1/KC mRNA expression was induced early (2 h) by LMLP. Increased expression of mRNA was parallel to increased CXCL1/KC secretion, which was observed as early as 2 h and peaked and reached a plateau by 12–24 h.
(Tian, 2011) [33]	Renal proximal tubular cells(HK2) from healthy and *Leptospira*-infected patients	Detergent extract of *L*. *santorosai* serovar Shermani (24–48 hrs)	q RT PCR Western blot Immunohistochemistry	NA	Fibronectin immunostaining intensity was markedly increased in the kidneys of patients with *Leptospira* infection compared to normal kidney tissues. Live serovar Shermani induced increased fibronectin production mainly through TLR2 and MyD88.
(Bernadi,2012) [50]	Lung tissues of patients who died because of leptospirosis	Live *Leptospira* (NR)	Immunohistochemistry	Increased TLR2 expression in the lungs of patients. Showed a sustained expression of TLR-2 in the endothelial cells of the small pulmonary vessels of patients who died of leptospirosis.	NA
(Zhang,2010) [29]	NRK52E cells (rat proximal tubule cells)	Cloned protein Loa22 (48hrs) *L*. *interrogans* Lai	q RT PCR	Compared with untreated NRK52E, TLR2 mRNA expression increased 0.8, 2.6, and 2.3 folds after incubation with Loa22 for 48 h at a concentration of 0.25, 0.5, and 1 mg/ ml, respectively.	Loa22 protein-induced iNOS and MCP-1 mRNA expression were completely inhibited by preincubation of TLR2-blocking antibody, but not by isotype control.
(Yijie,2016) [30]	BFF_NCC1 cells (Bovine fetal fibroblasts)	LPS of *L*. *interrogans* serovar hardjo (6–24 hrs) was extracted	q RT PCR	TLR2 mRNA expression level in L-LPS stimulated cells reached 1.9-fold compared with unstimulated cells. Up-regulation of TLR2 mRNA (relative fold 2–1) was completely inhibited by BMAP-28 compared to that of L-LPS stimulated cells. There was no effect on the L-LPS-induced TLR2 mRNA expression by replacing BMAP-28 with uBMAP- 28.	NA
			ELISA	NA	With the incubation of BMAP-28, enhancement of TNFα (200–37.5pg/mL) and IL-6(800-200pg/mL) was entirely inhibited compared to that of L-LPS- stimulated cells. When BMAP-28 was replaced with uBMAP-28, there was no effect on TNF-α and IL-6 mRNA expression induced by L-LPS.
(Bonhomme,2023) [40]	Bone marrow derived murine macrophage cell line (BMDMs)	*L*. *interrogans* Heat killed and Live (Manilae strain L495, Copenhageni strain Fiocruz L1-130, Icterohaemorragiae strain Verdun) *L*. *biflexa* (Patoc strain Patoc I)(24hrs)	Automated confocal microscopy analysis q RT PCR	NA	P62 accumulation is greatly impaired in TLR2-/-, TLR4-/- BMDMs (0–10%), increased in WT BMDMs (70–90%). P62 mRNA levels were increased in WT BMDMs (fold change 10) and abolished in TLR2-/-, TLR4-/- BMDMs (fold change 3).
(Inthasin,2023) [46]	Human kidney epithelium (HK2) cells	*L*. *interrogans* serovar Autumnalis(6hrs)	q RT PCR	The fold of TLR2 mRNA expression in *Leptospira*-infected HK2 cells did not differ from unstimulated HK2 cells.	mRNA expressions were significantly reduced in the anti hTLR2 pre-treated HK2 cells before being infected with *Leptospira* fold change- hBD2 (15–8), IL-1β (6–1.5), IL-6(5–1.5), IL-8(3.5–1.5), TNF-α (15–5).
(Novak,2022) [52]	Canine, mouse, and human TLR2 expressing HEK Blue reporter cells	Chemically inactivated pathogenic L. interrogans Canicola, Icterohaemorrhagiae, Australis, L. kirschneri Grippotyphosa (24hrs)	Absorption at 630/650 nm	Significantly activated canine, mouse, and human TLR2-expressing reporter cells in a dose-dependent manner.	NA
(Varma,2023) [49]	Murine macrophage cell lines hTLR2 (HEK-TLR2) THP1 macrophages pre-blocked with TLR2 monoclonal anti body	L-LPS(24 hours) Icterohaemorrhagiae strain RGA (R-LPS), Pomona (P-LPS), Hardjo (H-LPS), and from non-pathogenic L. biflexa serovar semeranga strain Potac1 (S-LPS)	ELISA ELISA ELISA	NA NA NA	TLR2−/− macrophages produced a significant level of IL-6 and TNF-α. IL6 (H-LPS-300pg/mL, P-LPS-190pg/mL, R-LPS-100pg/mL, S-LPS-400pg/mL). TNF-α (H-LPS-700pg/mL, P-LPS-400pg/mL, R-LPS-250pg/mL, S-LPS-800pg/mL). TLR4−/− and DKO macrophages failed to induce these cytokines. Produced significant levels of IL8 (H-LPS-650pg/mL, P-LPS 800pg/mL, R-LPS- 500pg/mL, S-LPS-850pg/mL). Reduced the secretion of IL6 and TNF-α. IL6 (H-LPS ~200 - ~100pg/mL,P-LPS ~200 - ~100pg/mL,R-LPS ~375- ~100pg/mL,S-LPS ~400-~100pg/mL) TNF-α(H-LPS ~350 - ~100pg/mL, P-LPS ~350 - ~100pg/mL, R-LPS ~600 - ~100pg/mL, S-LPS ~750 - ~100pg/mL)

Abbreviations: mRNA-messenger Ribonucleic acid, TLR2-Toll-like receptor 2, NR- Not reported, NA-Not applicable, TLR4-Toll-like receptor 4, TLR5-Toll-like receptor 5, LipL32- *Leptospira* outer membrane protein, DKO-Double knockout mouse, RT qPCR-Reverse transcriptase qPCR, WT- Wild type, L LPS-LPS of *Leptospira* spp., IL6-Interleukin 6, TNFα-Tumor necrosis factor α, CCL2/MCP1- Monocyte chemoattractant protein 1, IL8-Interleukin 8, OMPs-Outer membrane proteins, Lsa 21-*Leptospira* surface adhesive 21, rLoa 22-LPGN- *Leptospira* outer membrane protein A like protein-*Leptospira* peptidoglycan complex. siRNA-small interfering RNA, CXCL1/KC-Keratinocyte derived chemokine, Myd88-Myeloid differentiation factor 88, hBD2-human beta defensing 2, BMAP28- Bovine myeloid antimicrobial peptide 28, TRAF6- Tumor necrosis factor receptor-associated factor 6.

**Table 2:** *Ex-vivo* study characteristics.

ID	Cell line /Tissue(Ex-vivo)	Stimulant & incubation period	Analysis & sample storage	Main finding	
				Direct Response	In-direct Response
(Rajeev, 2020) [45]	Canine whole blood	*L*. *interrogans* Copenhageni *L*. *biflexa* Patoc (18 hrs)	RT2 profiler PCR array (Cell pellets in liquid Nitrogen)	The upregulated TLR2 gene (fold change TLR2-20.30) was observed in all of the individual canine blood samples upon stimulation with *L*. *interrogans* and *L*. *biflexa* compared to controls.	NA
(Inthasin,2018) [47]	Human oral tissues	*L*. *interrogans* serovar Autumnalis (4hrs)	q RT PCR (cDNA at -20°C)	NA	Expression (fold change) of pro-inflammatory cytokine IL-1β (8 to 3), IL-8(32 to 8), TNF-α (9 to 4), and hBD2 (12 to 2) were significantly reduced in the presence of anti -hTLR2 Ab.
(Charo, 2019) [55]	Healthy human blood	Fiocruz L1–130 of *L*. *interrogans* (3-18hrs)	ELISA (NR)	NA	Increased expression of IL‐8(1200 to 750 pg/mL) induced by Patoc or LIC was suppressed in neutrophils that were previously treated with an anti‐TLR2, but not with an anti‐TLR4.

Abbreviations: PCR-polymerase chain reaction, TLR2- Toll-like Receptor 2, TLR4-Toll-like Receptor 4, RT qPCR-Reverse transcriptase quantitative PCR, cDNA-complementary DNA, IL 1β- Interleukin 1β, IL8- Interleukin 8, TNFα- Tumor necrosis factor α, hBD2-human beta defensin 2, ELISA-Enzyme linked immunosorbent assay, LIC- *L*. *interrogans* Copenhageni, NR-Not reported, NA-Not applicable.

### TLR2 response in *In-vivo* studies

#### Direct response

Even though TLR4 is the most defensive PRR in mice/hamster models [12, 44] against leptospirosis, TLR2 contributed to triggering the immune response. Differential expression of the TLR2 gene was observed in mice renal cells upon the infection of *L*. *interrogans* Copenhageni [37]. Inducible effect of ‘Agonist’ components like *E*. *coli* LPS [18], Iris polysaccharide [31], and Pam3CSK4 [19] on TLR2 followed *Leptospira* infection increased the TLR2 expression in hamsters [18, 31] and mice [19] models compared with infected controls. TLR2 involvement in the pathological process of *Leptospira* infection was detected in Mice [37, 44] and Syrian golden hamsters [18, 31]. Double knockout mice (DKO)(TLR2-/-,TLR4-/-) were very susceptible to infection, while all the wild-type mice(WT) and TLR2 -/- mice survived [44].

#### Indirect response

Mice models deficient for TLR2 showed reduced levels of TNFα, IL6 [20] and reduced mRNA of IFNγ, iNOS [44] compared with wild-type mice. ‘Agonist’ activation of TLR2 increased the expression of IL1β, TNFα in mice kidney, liver, and lungs [31]. Moreover, TLR2 ‘Agonist’ Pam3CSK4 improved the ratio of IL10/ TNFα which known to be protective against infection [19]. TLR2 knockdown Zebra fish larvae demonstrated reduced kidney injury compared with wild-type zebra fish upon LipL32 inoculation [38]. TNFα, IL6, IL1β, IFNγ, iNOS, and IL10 expressed and/or secreted both in *in-vitro* and *in-vivo* studies with the involvement of TLR2. Diverse findings on TLR2-dependent IL6 and TNFα secretion, observed in an original study [44] and narrative review [59] showed increased IL6 and TNFα mRNA levels in DKO mice compared with WT mice. This observation implies, that other PRRs apart from TLRs are responsible for IL6 and TNFα production. Table 3 shows the characteristics of the *in-vivo* studies.

**Table 3:** *In-vivo* study characteristics.

ID	Animal model	Stimulant & incubation period	Analysis and sample storage time	Main finding	
				Direct TLR2 response	Indirect TLR2 involvement
(Chassin, 2009) [44]	Female C57BL/6/J mice	*L*. *interrogans* Fiocruz L1–130 (3 days)	DNA qPCR	NA	DKO mice (TLR2-/-, TLR4-/-) were very susceptible to infection and died 4–6 days post infection, while all the WT mice and tlr2 -/- mice survived. Bacterial load in WT mice tissues rapidly declined after 3 days. DKO bacterial load progressively increased 3 days of infection.
			q RT PCR Infectious organ tissues	NA	Low IFN-Γ (Liver-1.5, kidney-7.5, Lung-0.5) and iNOS (Liver-0.5, kidney-2, lung 7.5) were detected in tlr2-/- mice compared with WT mice. High mRNA for pro-inflammatory cytokines IL-6(Liver 15, kidney 5), TNF (Liver and kidney 50), and chemokine RANTES (liver-100, kidney 20), MIP-2(liver 250, kidney-5) were observed in tlr2-/- mice compared with WT mice.
			Plasma biochemical analysis (plasma stored at -80 C)	NA	Blood urea nitrogen, serum creatinine, serum bilirubin, and aspartate aminotransferase activity were significantly higher in DKO mice compared with WT. Levels of serum bilirubin and serum creatinine were also significantly higher in infected tlr2- /- and tlr4 -/- compared with infected WT mice.
(Chang, 2016) [38]	Zebra fish larvae	*L*. *santarosai* serovar Shermani strain LT821 (NR)	q PCR (lip32 & flaB), Immunohistochemistry, Whole-mount in situ hybridization	NA	Ectopic expression of LipL32 in the kidney—> inducing inflammation and mislocalization of NA-K-ATPase—>kidney injury. Ectopic expression of lipl32 mRNA triggers the accumulation of l-plastin positive cells in the posterior blood island and the region surrounding pronephric ducts. The inflammatory response was significantly abolished (P < 0.0001) in the tlr-2 knockdown larvae but remained unchanged with concomitant tlr4a and tlr4b knockdown (morphological deformities of the kidney were observed using fluorescence microscopy).
(Chou, 2018) [37]	C57BL/6 female mice	*L*.*interrogans* Copenhageni Fiocruz *L*. *biflexa* (7–28 days)	Microarray analysis and RNA sequencing	At day 7 post-infection with a pathogen, DEGs (Differentially expressed genes) were significantly enriched in TLR signaling pathways. This analysis revealed that 26 immune related genes such as LBP, FCGR1, SYK, IL33, COLLA1, IRF7, NCF1, and TLR2 in the *L interrogans* infected renal transcriptome were differentially expressed.	
(Zhang, 2020) [18]	Syrian golden hamsters	*L*. *interrogans* serovar Lai	q RT PCR (NR)	Expression levels of TLR2 were higher in the *E*. *coli* LPS-treated group than in infected controls. Fold change Kidney- 2, Liver- 35, Lungs- 3	Expression of both pro-inflammatory factors, TNF-α (kidney-4.5, Liver-3, Lung-2) and IL-1β (kidney-16, Liver-35, Lung-8), and anti-inflammatory IL-10(kidney-2, Liver-10, Lung-5) were elevated after treatment with *E*. *coli* LPS compared with infected controls.
(Liu, 2021) [31]	Syrian golden hamsters	*L*. *interrogans* serovar Lai strain Lai (56601), (4 days)	q RT PCR (NR)	Gene expression (fold induction-fi) of TLR2 in the kidney (60–100) and lung (5–12) was significantly increased compared to the infected controls. TLR4 was significantly improved only in the Liver.	Elevated fold induction was observed for IL1β- Liver (400–800), Lung (8–22), and TNFα-kidney (30–50), Lung (3–6).

Abbreviations: DNA qPCR-DNA quantitative Polymerase Chain Reaction, mRNA-messenger Ribonucleic acid, TLR2-Toll-like receptor 2, NR- Not reported, TLR4-Toll-like receptor 4, LipL32- *Leptospira* outer membrane protein, DKO-Double knockout mouse, RT qPCR-Reverse transcriptase qPCR, WT- Wild type, LPS-Lipopolysaccharide, IFNγ- Interferon γ, iNOS- Inducible Nitric oxide synthase, IL6-Interleukin 6, TNFα-Tumor necrosis factor α, MIP2- Macrophage inflammatory protein 2, IL1β- Interleukin 1β, IL10-Interleukin 10.

### TLR2 response in combined studies of human, *in-vivo* and *in-vitro*

Leptospirosis confirmed human serum samples were used to assess pro and anti-inflammatory cytokines [17] and circulatory micro RNAs [56]. As animal models, mice were used in both studies to assess cytokine levels and micro RNAs during leptospirosis. Infectious agents of mice were *L*. *interrogans* serogroup Icterohaemorrhagiae Lai [17] and LPS of *L*. *interrogans* Automnalis strain N2 [56]. THP-1 cells were used in both *in-vitro* studies. Recombinant hemolysin proteins [17] and LPS of *Leptospira* spp. [56] were used as infectious agents. TLR2 involvement during the pathogenesis response against leptospirosis was observed through the decrease of IL1β, TNFα [17, 56], IL6 [17], NFκB, and IL10 [56] secretion/expression due to deficient/knocked down of the TLR2 receptors [17, 56]. In addition to TLR2 involvement, TLR4 involvement was also observed during the infection [17]. Table 4 shows the characteristics of combined studies.

**Table 4:** Combined study characteristics.

ID	Cell line / Animal model / Patient sample	Stimulant & Incubation period	Analysis & sample storage	Main findings(In direct TLR2 response)
(Wang,2012) [17] ***	THP-1 or J774A.1 Mouse monocytes	rL-hemolysin proteins (rSph1, rSph2, rSph3, rHlpA and rTlyA) (24 h at 37˚C).	ELISA (cell supernatant) NR	TLR2 or TLR4-IgG significantly inhibited the IL-1β, IL-6, TNF-α production, and combining both TLR2-IgG and TLR4-IgG provided stronger inhibition. TLR2-/-, TLR4-/- showed significantly lower levels of IL-1β, IL-6 and TNF-α. Double-deficient mouse cells showed no cytokine-level response. TLR1, TLR5, and TLR6 signaling are not required for cytokine production.
	C3H/HeJ mice, Female C57BL/6 mice	*L*. *interrogans* serogroup Icterohaemorrhagiae Lai (48 hrs)	Protein Micro Array (serum) NR	Much less elevated 4 pro-inflammatory factors (IL-1β, IL-6, IL- 17, and TNF-α), an anti-inflammatory factor (IL-10), and two chemotactic factors (MCP-1 and RANTES) during the acute phase of infection.
	Patient serum (Observed by Dark-field microscopy, culture)	*L*. *interrogans* Lai Infection (within 3 days)	Protein Microarray (serum) NR	Among the sixteen elevated cytokines, eight were pro-inflammatory factors (IL-1β, IL-6, IL-17, and TNF-α) and anti-inflammatory factors (IL-4, IL-10, IL-13, and sTNF RI) colony-stimulating factors (G-CSF and GM-CSF), or chemotactic factors (MCP-1, MIP-1d, and EOTAXIN-2).
(Akino, 2020) [56] ***	THP-1 cells	LPS of *Leptospira* spp. Autumnalis strain N2 (3hrs)	RT qPCR Microarrays and miRNA profiling NR	TLR2 knockdown cells significantly inhibited the elevated cytokine mRNA levels. TNFα (95–10), NF-kB (16–2), IL-1β (6–0.1), and IL-10(150–5). 18 miRNAs were up-regulated. Knocking down TLR2 normalized the upregulated miRNA levels, indicating that these miRNAs are specific to the TLR2-LPS immune axis.
	10–12 week-old BALB/c mice	PBS or LPS of *Leptospira spp*.	qRT PCR (mice serum) NR	mRNA levels TNF-α, NF-kB, IL-1β, and IL-10 increased within 3 h of LPS stimulation. Of the miRNAs upregulated at day 4 or day 7, three (miR-21-5p, miR-144-3p, and miR-let-7b-5p) shared similarities with the human miRNome profile of the THP1 cells exposed to LPS of *Leptospira* spp.
	Patient serum samples	*Leptospira* spp. infection (0–10 days)	RT-qPCR (serum) NR	The fold changes of circulating miR-21-5p, miR-144-3p, and miR-let-7b-5p in the serum of confirmed cases of leptospirosis were significantly higher (P 0.001) than those in healthy controls and persons diagnosed with other febrile illness.
(Werts,2001) [20] **	THP-1 cell line	LPS of *L*. *interrogans* Icterohaemorrhagiae strain Verdun or Intact *L*. *interrogans*(6h)	ELISA (Cell supernatants) NR	Cytokine inhibition was resulted by blocking hTLR2 but not anti hTLR4. TNFα (~2 - <0.5 ng/mL), IL8(~3.5–1.5 ng/mL)
	C57BL/6 mice	LPS of *Leptospira* spp. (90 min)	ELISA(Cell supernatant) NR	Within 24hrs WT mice showed acute symptoms and died. TLR2-deficient mice showed no symptoms and survived. The serum of wild-type mice contained high amounts of TNF-α (8-12ng/mL) and IL-6(4-6ng/mL). In contrast, TLR2-deficient mice produced neither TNF-α (0-4ng/mL) nor IL-6(0-2ng/mL) in response to LPS of *Leptospira* spp.
(Viriyakosol, 2006) [16]**	Mouse peritoneal macrophages	LPS of *L*. *interrogans* (16h)	ELISA (Cell supernatant) NR	LPS of *Leptospira* spp. induced a much higher level of cytokine responses from WT murine macrophages (IL6-TNF-18000pg/mL) than from macrophages from TLR2-/- mice (IL6-2500, TNFα-5000pg/mL). TLR4 deficient macrophages failed to secrete MIP, TNFα, and IL6 compared with WT mice.
(Nahori,2005) [12]**	RAW264.7-Mouse macrophage cells	*L*. *interrogans* Icterohemorragiae strain Verdun LPS of *Leptospira* spp. & Lipid A were prepared (18h)	ELISA (Cell supernatant)	Response to whole LPS of *Leptospira* spp. was dramatically decreased in TLR2-/- mice (IL6 25000—>5000pg/mL), whereas the response in TLR4-/- was only partially diminished.
(Zhang,2016) [19]**	Hamster peritoneal macrophages	L. *interrogans* Autumnalis (24h)	qRT PCR (cell RNA extraction) NR	TLR2 and TLR4 did not influence TNFα levels. TLR2 agonist Pam3CSK4 improved IL-10 but not TNFα levels in peritoneal macrophages from hamsters. This result explains the improved IL-10/TNFα ratio in hamsters treated with Pam3CSK4. Reduced IL-10/ TNFα ratio was observed in TLR2-/-mice compared with WT mice.
(Santecchia,2019) [43]**	Human monocytes, Mice macrophages	*L*. *interrogans* Manilae,Verdun,Fiocruz(24 hrs)	ELISA (Cell supernatant) NR	Marked reduction of bacterial burden in TLR2 agonist (CL429) treated mice compared with untreated mice. Increased IL6 (~4000pg/mL), NO (~1μM), KC (1500-2500pg/mL), and RANTES (15000pg/mL) were observed in mice bone marrow-derived macrophages treated with CL249 compared with untreated cells. Increased IL6 (~10000pg/mL) and RANTES (~1500-2500pg/mL) were observed in mice peritoneal macrophages.

Abbreviations: miRNA-micro Ribonucleic acid, TLR-Toll-like receptor, NR- Not reported, DKO-Double knockout mouse, RT qPCR-Reverse transcriptase qPCR, WT- Wild type, L LPS-LPS of *Leptospira* spp., IL-Interleukin, TNFα-Tumor necrosis factor α, CCL2/MCP1- Monocyte chemoattractant protein 1, RANTES- Regulated upon Activation, Normal T Cell Expressed and Presumably Secreted. *** Human + In-vivo + In-vitro, ** In-vivo+ In-vitro, ** In-vivo+ Ex-vivo.

### TLR2 response in combined studies of *in-vivo* and *in-vitro*

Four studies were combined with *in-vivo* and *in-vitro* experimental models (n = 4) (Table 4). The prominent animal models were mice [12, 16, 20, 43] and Syrian golden hamsters [19]. Predominantly used cell lines were mouse macrophages [12, 16], human peripheral blood monocytes [43] and THP-1 [20]. The most used infectious agent was LPS of *Leptospira* spp. [16]. Other inoculated organisms were *L*. *interrogans* Manilae, Verdun, Fiocruz [43], and *L*. *interrogans* Autumnalis [19]. TLR2 involvement was observed through inhibition/reduction level of secretion/expression of TNFα [16, 20], IL6 [12, 16, 20], IL8 [20], and IL10 [19] in TLR2 deficient/blocked cells compared with intact cells. TLR4 involvement during the infection was also observed through mediating IL6 [12, 16] and TNFα [16]. With regard to survival, LPS treated wild type (WT) mice showed acute illness and death within 24 hours; in contrast, TLR2 deficient mice showed no shock and survived [20].

### TLR2 response in combined studies of *ex-vivo* and *in-vivo*

Mice were used as animal models, while mice peritoneal macrophages were used as *ex-vivo* cell lines in one study [43] (Table 4). *L*. *interrogans* Copenhageni and *L*. *interrogans* Manilae were used as infectious agents [43]. TLR2 Agonist, CL429 treated human monocytes and mice peritoneal cells showed increased levels of IL6 and NO, IL1β respectively compared with CL429 untreated cell lines.

### TLR2 response in human studies

Whole blood samples from the confirmed patients with leptospirosis were used in all three human studies (n = 3). Microscopic Agglutination Test (MAT) and Polymerase Chain Reaction (PCR) were used as the disease confirmatory test. Significantly increased TLR2 expression was observed on polymorphonuclear cells [42] and neutrophils [51] of *Leptospira*-infected human whole blood (n = 30) compared with healthy human whole blood. Contrary to that, *Leptospira*-infected human monocyte (n = 57) TLR2 expression did not show a significant difference compared with healthy human whole blood [41]. In human and *in-vivo* studies, TLR2 response was observed within 7 days of infection, which is a longer period than that observed in *in-vitro* studies (2 days). Table 5 shows the characteristics of human studies.

**Table 5:** Human study characteristics.

ID	Leptospirosis confirmation test	Infected organism & incubation period	Analysis & sample storage time period	Main Findings (Direct TLR2 response)
(Raffray, 2016) [42]	PCR- 23S r RNA IgM- ELISA (>50 U/mL), MAT >1/400	*L*. *interrogans* Icterohaemorrhagiae, Canicola, Ballum (1–7 days)	Flow cytometry (whole blood) <6 hrs	Significantly increased (p = 0.001) the TLR2 expression on PMN in the leptospirosis group (n = 15) (MFI = 0.92) compared with healthy control (n = 13) (MFI = 0.57).
(Raffray, 2019) [41]	PCR- 23S r RNA IgM-ELISA- (>50 U/mL) MAT >1/400 titer	*L*.*interrogans* Icterohaemorrhagiae, Canicola, Ballum (3–5 days)	Flow cytometry (whole blood) NR	The levels of TLR2, TLR4, and CD69 in monocytes were not significantly different between healthy (n = 37) and leptospirosis groups (n = 57). MFI values; TLR2 (H-10000 L<10000), TLR4 (H~2500 L~2500), CD69 (H-~2100 L-2000-2500).
(Lindow, 2019) [51]	PCR- 16s r RNA, LipL32 MAT > 1/800	*Leptospira* sp. Infection (1–7 days)	Flow cytometry (whole blood) <4hrs	TLR2 expression was significantly higher on neutrophils from patients (n = 15) with more severe (9%) leptospirosis (lung or/& renal dysfunction) & acute (3%) (p<0.05) (Lacking organ dysfunction) relative to healthy controls (n = 4) (1%) (p<0.01). (%-percent granulocytes positive for TLR2)

Abbreviations: PCR-Polymerase Chain Reaction, r RNA-ribosomal Ribonucleic acid, IgM-ELISA- Immunoglobulin M-enzyme linked immunosorbent assay, MAT- Microscopic agglutination test, TLR2-Toll-like receptor 2, PMN- Polymorphonuclear, MFI- Mean fluorescence intensity, NR- Not reported, TLR4-Toll-like receptor 4, LipL32- *Leptospira* outer membrane protein.

### Other TLR responses during *Leptospira* infection

Among 35 articles, some of the studies demonstrated the response of TLR4 [12, 14–19, 31] and TLR5 [15] along with the TLR2 response during the infection of *Leptospira* spp./cell components. IL6 [12, 14, 15, 17], IL-1β [14, 17], TNFα [14, 17], MIP [16], CCL2 [14], CCL10 [14], COX2 [14], MCP1 [14], IFNγ [14], iNOS [14] were identified as reduced/downregulated immune mediators in cell lines of deficient/knocked down TLR4 compared with intact cell lines. TNFα reduction was also observed in cells that blocked TLR5 [15]. Animal studies showed TLR4 involvement in triggering inflammatory mediators such as IL-1β [18, 31], TNFα [18, 31], IL10 [18], NO [18], iNOS [18], anti *Leptospira* IgG [18]. The contribution of both TLR4 and TLR2 in leptospirosis pathogenesis was observed in DKO mice by showing dramatically low mRNA expression of IFNγ and iNOS, compared with WT mice [44].

### Risk of bias in studies

In OHAT risk of bias assessment, all *in-vitro* and *ex-vivo* studies (27 of which used cell lines) present a ’fair’ risk of bias rating. Cell line experiments present a ’definitely low’ risk for selection bias, but blinding of experimental personnel is ’not reported’, and performance bias is ’probably high’. There is a ’definitely low’ risk of bias attrition and detection bias in all studies. All studies present ’low’ selective reporting bias and other biases. SYRCLE’s risk of bias assessment for 12 in-vivo studies shows a similar rating with an ’unclear’ risk of bias in 2 domains of selection bias. All studies have an ’unclear’ risk of performance and detection bias, as there is no report of randomly housed or blinded intervention and no animal random selection for outcome assessment or blinded outcome assessors. No studies have attrition bias, reporting bias, or other biases. In all 5 human studies, NIH-developed quality assessment was used with a similar risk of bias rating. Sample size justification, random selection of patients, and concurrent control selection were not reported, leading to selection and performance bias. Potential confounding variable analyses were not reported, contributing to another risk of bias. S5–S7 Tables show the risk of bias results for each study.

### Certainty of evidence

Five domains—risk of bias, inconsistency, indirectness, imprecision, and publication bias—were used to assess the certainty of outcomes in *in-vivo* and human studies (S2 Appendix). Risk of bias was assessed and reported for individual studies but not rated down for inherent limitations. Inconsistency was not considered as no meta-analysis was performed, and the direction of estimates did not vary. Indirectness was not down-rated, as all studies matched the PICO criteria. Imprecision was down-rated as no studies had sample size justification. Publication bias was not down-rated as no conflicts of interest were present. GRADEpro generated Certainty of the outcome, and ’increased TLR2 expression during leptospirosis’ in human and *in-vivo* studies was rated as ’Moderate.’ Table 6 shows the certainty of outcomes from *in-vivo* and human studies.

**Table 6:** Certainty of outcomes from *in-vivo* and human studies.

Certainty assessment							Impact	Certainty
Study ID	Study design	Risk of bias	Inconsistency	Indirectness	Imprecision	Other considerations		
(Chassin,2009) (Chou,2018)	observational studies	not serious	not serious	not serious	serious^a^	none	TLR2 involvement against *L*.*interrogans* infection was observed.	⨁⨁⨁◯Moderate
(Zhang,2020) (Liu, 2021)	observational studies	not serious	not serious	not serious	serious^a^	none	TLR2 expression was increased compared with uninfected controls. *E*. *coli* Lipopolysaccharide and Iris polysaccharide treated prior to infection.	⨁⨁⨁◯Moderate
(Chang,2016)	observational studies	not serious	not serious	not serious	serious^a^	none	The inflammatory response against *L*. *santorosai* was significantly abolished in TLR2 knockout zebra fish larvae, but unchanged for TLR4 knockout.	⨁⨁⨁◯Moderate
(Raffray 2016) (Lindow,2019)	observational studies^a^	not serious	not serious	not serious	serious^b^	none	TLR2 expression was significantly increased on polymorphonuclear neutrophils in the leptospirosis group compared to healthy individuals.	⨁⨁⨁◯Moderate
(Raffray,2019)	observational studies^a^	not serious	not serious	not serious	serious^c^	none	No significant difference in TLR2 expression on Monocytes between groups of healthy and leptospirosis.	⨁⨁⨁◯Moderate

a. Not detected specific study design. In terms of experimental design, they used confirmed leptospirosis cases and healthy controls. b. Not detected sample size justification. However, a total of 30 patient samples and 17 healthy controls were used. c. Not detected sample size justification.

## Discussion

This systematic review identifies the role of TLR2 during the infection of pathogenic *Leptospira* spp. or its’ cell components by extracting the data from 35 original studies. Although, the review yielded valuable data on the role of TLR2 during leptospirosis, there were equivocal findings on TLR2 direct response.

We observed increased TLR2 gene expression in non-human cell lines [29, 30, 32, 39, 45, 53, 54], human polymorphonuclear cells [42], human neutrophils [51], and lung tissues obtained from patients who died because of leptospirosis [50]. Conversely, we found TLR2 expression did not differ in human monocytes and HK2 cells during *Leptospira* infection [41, 46]. The contention, that TLR2 is the predominant PRR for gram-positive and other bacterial products that are distinct from gram-negative LPS [4, 60] may address the controversial TLR2 expression during leptospirosis even among human/human cell line studies.

LPS of *Leptospira spp*. (L LPS) has a unique structure to other gram-negative bacterial LPS [61], while L LPS is not recognized through human TLR4, but is recognized by mice TLR4 that mice immune response is adapted to *Leptospira* spp. infection [12]. Nevertheless, TLR2 activation was also observed in mice proximal tubule epithelial cells through *L*. *santerosai* Shermani inoculation [39]. Increased TLR2 expression in mice and hamster models was observed in response to *Leptospira* LipL32 as well. Agonist Pam3CSK4 triggered immune response via TLR2, increasing hamster survival rate and reducing organ lesions [14, 15, 19, 33, 34, 55]. Besides mice/hamster models, pig, and bovine cells responded to LPS of *Leptospira spp*. via TLR2 [32, 53, 54]. Aligned with our findings, narrative reviews discussed the renal dysfunction associated with leptospirosis that resulted in secretion/expression of Fibronectin [62], iNOS [62–66], CCL2/MCP-1 [62, 63, 65, 66], TNFα [62, 64–66], NFκB [62, 67], CCL2/MIP2 [66], CXCL1/KC [66] through TLR2.

A previous systematic review [68] showed elevated levels of cytokines (IL-1β, IL-2, IL-4, IL-6, IL-8, IL-10, TNFα) in severe human leptospirosis compared with mild leptospirosis. Since those studies did not explore the TLR2 involvement in triggering immune responses, they did not meet our inclusion criteria. As per our findings, recognition of PAMPs by the host TLR2 triggers the activation of immune effectors such as cytokines/chemokines IL6 [12, 14–17, 20, 32, 43, 46, 49], IL8 [20, 36, 46, 47], IL 1β [14, 17, 43, 46, 47, 56], TNFα [14–17, 20, 32, 36, 46, 47, 49, 56], IFNγ [14], IL10 [19], CCL2/MCP-1 [14, 29, 34, 36, 39], CCL10 [14, 34–36], COX2 [14], CXCL1/KC [34], and CXCL2/MIP2 [35] followed by leukocyte recruitment to heal tissues/organ lesions. While clearing bacteria, antimicrobial peptides hBD2 [46, 47], iNOS [29, 39], Fibronectin [33], P62 [40], and Oxygen, Nitrogen reactive species are produced [18, 44]. Although inflammatory response is defensive against the bacteria, excessive levels can cause harmful effects such as organ lesions [19, 38]. To determine whether innate TLR2 immunity has a protective or harmful effect, it is crucial to recognize the level and threshold of pathogen-host immune responses, thereby accurately interpreting the role of TLR2. As per our results, the TLR2 activator (Agonist) and the TLR2 blocker (Antagonist) can be used to modulate the level of immune effectors and enhance the immune response against the infection. Another crucial factor in identifying the TLR2 response is the incubation period of the bacterium in the host tissues or host body. *In-vitro* studies observed TLR2 mRNA expression within 48hrs of infection, while human and *in-vivo* studies showed TLR2 response no longer than 7 days after the infection.

Nevertheless, the studies on other bacterial infections demonstrated the downregulation of TLR2 gene via different types of mechanisms. Kim and the colleagues [69] demonstrated that TLR2 downregulation may be associated with patient mortality in the early stage of *Staphylococcus aureus* bacteremia (SAB). Several other studies on *Staphylococcus aureus* have also demonstrated the human and mouse TLR2 inactivation during the bacterial infection [70–72].

This process is mediated through Staphylococcal superantigen- like protein (SSL3) that directly binds to the extracellular domain of TLR2 and inhibits TLR2 activation on human and murine monocytes, macrophages, and neutrophils [73]. Micro RNAs (miRNAs) do not encode proteins, but regulate the gene expression in humans [74],by base pairing to the 3’ untranslated region (UTR) of the target gene mRNA [75]. Syphilis is caused by the bacterium *Treponema pallidum*, and the infection has up-regulated the human Micro RNA (miR-101-3p) that paired with the 3’ UTR of mRNA of the TLR2 gene to downregulate the TLR2 gene expression and eventually reduced the cytokine secretion [76]. Virus itself can express Micro RNA s during human infection, as miR-UL112-3p has been demonstrated as a human TLR2 downregulator that inhibits the downstream cascade of reactions, to evade the antiviral immune responses [77]. Whether a similar mechanism exists in *Leptospira* spp. infection is yet to be investigated.

Due to the diversity of the studies in deploying different types of populations, *Leptospira* whole organism/cell components, different analysis methods, and direct/indirect TLR2 response, it is challenging to make sense of the role that TLR2 plays during human leptospirosis. Concerning the methods, Flow cytometry, q RT PCR, Microarray analysis and RNA sequencing were deployed to assess the direct TLR2 response that would slightly affect the contrast findings since those are robust enough to determine the direct TLR2 response. Nevertheless the pathogenesis differences of the *Leptospira* spp. and the cell components could be attributed to the immune response differences[78]. Further, we observed increased TLR2 direct response in non-human studies [29, 30, 32, 39, 45, 53, 54], regardless of the type of the cell line or the animal model used, except equivocal findings observed in human cell line/ human studies [41, 42, 46, 50, 51]. Answering the question on the role of TLR2 during leptospirosis makes it challenging to anticipate how accurately these findings reflect in humans due to the utilization of diverse cell lines and experimental animal models that would not show the real synergistic effect of the human body. Also it is worth knowing the real time TLR2 expression after the onset of *Leptospira* exposure, which was lacking in human studies and can be controlled in *in-vivo*, *in-vitro* and *ex-vivo* studies. The main limitation observed is the limited number of human studies identified during the search. Our review was limited to research published in English medium and this could have contributed to this limitation slightly. Rather than focusing on conducting *in-vivo*, *in-vitro*, and *ex-vivo* studies it is beneficial to conduct human studies to establish a solid evidence of human TLR2 response. As a future research direction, it is worth collecting the patient samples to observe the response of the direct TLR2 at specific time points after the infection of *Leptospira* spp., though it is difficult to acquire those samples in a clinical setup. Results of human TLR2 expression during the period of *Leptospira* spp. infection would be crucial in developing a vaccine candidate for the infection. Concerning the strengths, even with the inherent study qualities, human, *in-vivo*, *in-vitro*, and *ex-vivo* studies present an overall risk of bias rating as ‘Fair’, ‘Fair’, ‘Good’ and ‘Good’ respectively. GRADE approach assessed the important outcomes of *in-vivo* and human studies as ‘Moderate’ certainty. To our knowledge, this study is the first systematic review addressing the role of TLR2 during leptospirosis.

## Conclusions

Direct TLR2 expression against human leptospirosis is inconclusive due to ambiguous findings even in existing human studies. Also, the scarcity of human studies hinders the establishment of a robust evidence base for interpreting the direct response of human TLR2 during leptospirosis. It is essential to conduct further human studies to address the ambiguous findings of human TLR2 expression during leptospirosis. However, it has been observed that increased TLR2 expression and the secretion/mRNA expression of various cytokines/chemokines (IL6, IL8, IL 1β, TNFα, IFNγ, IL10, CCL2/MCP-1, CCL10, COX2, CXCL1/KC, CXCL2/MIP2) and immune effectors (antimicrobial peptides hBD2, iNOS, Fibronectin, Oxygen, and Nitrogen reactive species) are significant functions of TLR2 in leptospirosis. While an immune response against the bacterium is crucial for overcoming the disease, the subsequent detrimental effects of excessive immune mediators on tissues/organs cannot be ignored. The findings of this systematic review provide valuable insights for developing new therapeutic strategies aimed at maintaining a moderate level of immune mediators through TLR2 receptor agonists or antagonists. This systematic review emphasizes the need for human studies on TLR2 receptor expression /involvement during *Leptospira spp*. infection to develop new therapeutic strategies as vaccine candidates through TLR2.

## Other information

### Registration and protocol

International prospective register of systematic reviews.

PROSPERO 2022 CRD42022307480 (No amendments were provided at registration)

Available from https://www.crd.york.ac.uk/prospero/display_record.php?ID=CRD42022307480

The protocol was not prepared.

## Supporting information

S1 Table Search strings for the systematic review. (DOCX)

S2 Table General characteristics of the studies. (DOCX)

S3 Table PRISMA 2020 abstract checklist. (DOCX)

S4 Table PRISMA 2020 checklist. (DOCX)

S5 Table Risk of bias assessment. (DOCX)

S6 Table Risk of bias assessment. (DOCX)

S7 Table Risk of bias assessment. (DOCX)

S1 Appendix General characteristics of the studies. (XLSX)

S2 Appendix GRADE items. (XLSX)

## References

[1] Inada R, Ido Y, Hoki R, Ito H, Wani H. The serum treatment of weil’s disease (spirochiatosis icterohæmorrhagica). J Exp Med. 1916;24(5):485–96. 19868056 10.1084/jem.24.5.485 PMC2125472

[2] Stimson AM. Note on an Organism Found in Yellow-Fever Tissue. Public Heal Reports. 1907;22(18):541.

[3] Bharti AR, Nally JE, Ricaldi JN, Matthias MA, Diaz MM, Lovett MA, et al. Reviews Leptospirosis: a zoonotic disease of global importance. 2003;3(December):757–71. 10.1016/s1473-3099(03)00830-2 14652202

[4] Aderem A, Ulevitch RJ. Toll-like receptors in the induction of the innate immune response. 2000;406(August). doi: 10.1038/35021228 10963608

[5] Hashimoto C, Hudson KL, Anderson K V. The Toll gene of drosophila, required for dorsal-ventral embryonic polarity, appears to encode a transmembrane protein. Cell. 1988;52(2):269–79. doi: 10.1016/0092-8674(88)90516-8 2449285

[6] Medzhitov R, Preston-Hurlburt P, Janeway CA. A human homologue of the Drosophila toll protein signals activation of adaptive immunity. Nature [Internet]. 1997;388(6640):394–7. Available from: http://www.scmp.com/article/995826/widow-nepali-shot-pc-seeks-compensation doi: 10.1038/41131 9237759

[7] Vijay K. Toll-like receptors in immunity and inflammatory diseases: Past, present, and future. Int Immunopharmacol. 2018;59(January):391–412. doi: 10.1016/j.intimp.2018.03.002 29730580 PMC7106078

[8] Zähringer U, Lindner B, Inamura S, Heine H, Alexander C. TLR2—promiscuous or specific? A critical re-evaluation of a receptor expressing apparent broad specificity. Immunobiology. 2008;213(3–4):205–24. doi: 10.1016/j.imbio.2008.02.005 18406368

[9] Matsushima N, Tanaka T, Enkhbayar P, Mikami T, Taga M, Yamada K, et al. Comparative sequence analysis of leucine-rich repeats (LRRs) within vertebrate toll-like receptors. BMC Genomics. 2007;8:1–20. 17517123 10.1186/1471-2164-8-124 PMC1899181

[10] Ren S xi, Fu G, gao Jiang X, Zeng R. Unique physiological and pathogenic features of Leptospira interrogans revealed by whole-genome sequencing. 2003;422(April). doi: 10.1038/nature01597 12712204

[11] Werts C, Werts C, Tapping RI, Mathison JC, hsien Chuang T, Kravchenko V, et al. Leptospiral lipopolysaccharide activates cells through a TLR2-dependent mechanism. 2001;(May). doi: 10.1038/86354 11276206

[12] Nahori MA, Fournié-Amazouz E, Que-Gewirth NS, Balloy V, Chignard M, Raetz CRH, et al. Differential TLR Recognition of Leptospiral Lipid A and Lipopolysaccharide in Murine and Human Cells. J Immunol. 2005;175(9):6022–31. doi: 10.4049/jimmunol.175.9.6022 16237097

[13] Haake DA. Spirochaetal lipoproteins and pathogenesis. Physiol Behav [Internet]. 2000;176(5):139–48. Available from: doi: 10.1099/00221287-146-7-1491 10878114 PMC2664406

[14] Faisal SM, Varma VP, Subathra M, Azam S, Sunkara AK, Akif M, et al. Leptospira surface adhesin (Lsa21) induces Toll like receptor 2 and 4 mediated inflammatory responses in macrophages. Sci Rep. 2016;6(1):1–14. 27996041 10.1038/srep39530 PMC5172228

[15] Goris MGA, Wagenaar JFP, Hartskeerl RA, van Gorp ECM, Schuller S, Monahan AM, et al. Potent innate immune response to pathogenic leptospira in human whole blood. PLoS One. 2011;6(3):e18279. doi: 10.1371/journal.pone.0018279 21483834 PMC3069077

[16] Viriyakosol S, Matthias MA, Swancutt MA, Kirkland TN, Vinetz JM. Toll-like receptor 4 protects against lethal Leptospira interrogans serovar icterohaemorrhagiae infection and contributes to in vivo control of leptospiral burden. Infect Immun. 2006;74(2):887–95. doi: 10.1128/IAI.74.2.887-895.2006 16428731 PMC1360355

[17] Wang H, Wu Y, Ojcius DM, Yang XF, Zhang C, Ding S, et al. Leptospiral hemolysins induce proinflammatory cytokines through Toll-like receptor 2-and 4-mediated JNK and NF-κB signaling pathways. 2012;7(8). 10.1371/journal.pone.0042266 PMC3411626 22870312

[18] Zhang W, Xie X, Wang J, Song N, Lv T, Wu D, et al. Increased inflammation with crude E. coli LPS protects against acute leptospirosis in hamsters. Emerg Microbes Infect. 2020;9(1):140–7. doi: 10.1080/22221751.2019.1710435 31914888 PMC6968624

[19] Zhang W, Zhang N, Xie X, Guo J, Jin X, Xue F, et al. Toll-like receptor 2 agonist Pam3CSK4 alleviates the pathology of leptospirosis in hamster. Infect Immun. 2016;84(12):3350–7. doi: 10.1128/IAI.00708-16 27620721 PMC5116713

[20] Werts C, Tapping RI, Mathison JC, Chuang TH, Kravchenko V, Saint Girons I, et al. Leptospiral lipopolysaccharide activates cells through a TLR2-dependent mechanism. Nat Immunol. 2001;2(4):346–52. doi: 10.1038/86354 11276206

[21] Echchannaoui H, Frei K, Schnell C, Leib SL, Zimmerli W, Landmann R. Toll-like receptor 2-deficient mice are highly susceptible to Streptococcus pneumoniae meningitis because of reduced bacterial clearing and enhanced inflammation. J Infect Dis. 2002;186(6):798–806. doi: 10.1086/342845 12198614

[22] Hsu SH, Lo YY, Tung JY, Ko YC, Sun YJ, Hung CC, et al. Leptospiral outer membrane lipoprotein LipL32 binding on toll-like receptor 2 of renal cells as determined with an atomic force microscope. Biochemistry. 2010;49(26):5408–17. doi: 10.1021/bi100058w 20513152

[23] Green S. Cochrane Handbook for Systematic Cochrane Handbook for Systematic Reviews of Julian PT Higgins and Sally Green. 2008;

[24] Page MJ, Mckenzie JE, Bossuyt PM, Boutron I, Hoffmann C, Mulrow CD, et al. RESEARCH METHODS AND REPORTING The PRISMA 2020 statement: an updated guideline for reporting systematic reviews Systematic reviews and Meta-Analyses. 2021; 10.1136/bmj.n71 PMC8005924 33782057

[25] NHLBI. Study Quality Assessment Tools | NHLBI, NIH. Natl Hear Lung, Blood Inst [Internet]. 2013;1–59. Available from: https://www.nhlbi.nih.gov/health-topics/study-quality-assessment-tools

[26] Hooijmans CR, Rovers MM, de Vries R, Leenaars M, Ritskes-Hoitinga M, Langendam MW. SYRCLE’s risk of bias tool for animal studies. BMC Med Res Methodol. 2014;14(1):1–9. doi: 10.1186/1471-2288-14-43 24667063 PMC4230647

[27] Rooney A. Extending a risk-of-bias approach to address in vitro studies. Washington, USA Natl Toxicol Progr Off Heal Assess Transl. 2015;

[28] Granholm A, Alhazzani W, Møller MH. Use of the GRADE approach in systematic reviews and guidelines. Br J Anaesth. 2019;123(5):554–9. doi: 10.1016/j.bja.2019.08.015 31558313

[29] Zhang Y, Bao L, Zhu H, Huang B, Zhang H. OmpA-like protein Loa22 from Leptospira interrogans serovar Lai is cytotoxic to cultured rat renal cells and promotes inflammatory responses. Acta Biochim Biophys Sin. 2010;42(1):70–9. doi: 10.1093/abbs/gmp109 20043049

[30] Yijie GUO, Ding C, Zhang B, Jun XU, Meng XUN, Jiru XU. Inhibitory effect of BMAP-28 on Leptospiral lipopolysaccharide-induced TLR2-dependent immune response in bovine cells. Jundishapur J Microbiol. 2016;9(6). 10.5812/jjm.33926 PMC5013549 27635213

[31] Liu J, Xie X, Zhang W, Cao Y. Immune-enhanced effect of Iris polysaccharide is protective against leptospirosis. Microb Pathog. 2021;154:104855. doi: 10.1016/j.micpath.2021.104855 33757897

[32] Guo Y, Xu J, Wang L, Xu J. Leptospiral lipopolysaccharide-induced cytokine production is dependent on toll-like receptor 2 in bovine cells. Pak Vet J. 2016;36:280–5.

[33] Tian YC, Hung CC, Li YJ, Chen YC, Chang MY, Yen TH, et al. Leptospira santorosai Serovar Shermani detergent extract induces an increase in fibronectin production through a Toll-like receptor 2-mediated pathway. Infect Immun. 2011;79(3):1134–42. doi: 10.1128/IAI.01287-09 21173310 PMC3067514

[34] Hung CC, Chang CT, Chen KH, Tian YC, Wu MS, Pan MJ, et al. Upregulation of chemokine CXCL1/KC by leptospiral membrane lipoprotein preparation in renal tubule epithelial cells. Kidney Int. 2006;69(10):1814–22. doi: 10.1038/sj.ki.5000362 16625148

[35] Hung CC, Chang CT, Tian YC, Wu MS, Yu CC, Pan MJ, et al. Leptospiral membrane proteins stimulate pro-inflammatory chemokines secretion by renal tubule epithelial cells through toll-like receptor 2 and p38 mitogen activated protein kinase. Nephrol Dial Transplant. 2006;21(4):898–910. doi: 10.1093/ndt/gfi316 16339163

[36] Hsu SH, Chang MY, Lin SM, Ko YC, Chou LF, Tian YC, et al. Peptidoglycan mediates Leptospira outer membrane protein Loa22 to toll-like receptor 2 for inflammatory interaction: a novel innate immune recognition. Sci Rep. 2021;11(1):1–16. 33441663 10.1038/s41598-020-79662-8 PMC8115183

[37] Chou LF, Chen TW, Yang HY, Chang MY, Hsu SH, Tsai CY, et al. Murine renal transcriptome profiles upon leptospiral infection: implications for chronic kidney diseases. J Infect Dis. 2018;218(9):1411–23. doi: 10.1093/infdis/jiy339 29868892

[38] Chang MY, Cheng YC, Hsu SH, Ma TL, Chou LF, Hsu HH, et al. Leptospiral outer membrane protein LipL32 induces inflammation and kidney injury in zebrafish larvae. Sci Rep. 2016;6(1):1–12. 27278903 10.1038/srep27838 PMC4899798

[39] Yang CW, Hung CC, Wu MS, Tian YC, Chang CT, Pan MJ, et al. Toll-like receptor 2 mediates early inflammation by leptospiral outer membrane proteins in proximal tubule cells. Kidney Int. 2006;69(5):815–22. doi: 10.1038/sj.ki.5000119 16437059

[40] Bonhomme D, Santecchia I, Escoll P, Papadopoulos S, Vernel-Pauillac F, Boneca IG, et al. Leptospiral lipopolysaccharide dampens inflammation through upregulation of autophagy adaptor p62 and NRF2 signaling in macrophages. Microbes Infect. 2023 Dec;105274. doi: 10.1016/j.micinf.2023.105274 38081475

[41] Raffray L, Giry C, Vandroux D, Fayeulle S, Moiton MP, Gerber A, et al. The monocytosis during human leptospirosis is associated with modest immune cell activation states. Med Microbiol Immunol. 2019;208(5):667–78. doi: 10.1007/s00430-018-0575-9 30542761

[42] Raffray L, Giry C, Vandroux D, Kuli B, Randrianjohany A, Pequin AM, et al. Major neutrophilia observed in acute phase of human leptospirosis is not associated with increased expression of granulocyte cell activation markers. PLoS One. 2016;11(11):e0165716. doi: 10.1371/journal.pone.0165716 27802348 PMC5089758

[43] Santecchia I, Vernel-Pauillac F, Rasid O, Quintin J, Gomes-Solecki M, Boneca IG, et al. Innate immune memory through TLR2 and NOD2 contributes to the control of Leptospira interrogans infection. PLoS Pathog. 2019;15(5):e1007811. doi: 10.1371/journal.ppat.1007811 31107928 PMC6544334

[44] Chassin C, Picardeau M, Goujon JM, Bourhy P, Quellard N, Darche S, et al. TLR4-and TLR2-mediated B cell responses control the clearance of the bacterial pathogen, Leptospira interrogans. J Immunol. 2009;183(4):2669–77. doi: 10.4049/jimmunol.0900506 19635914

[45] Rajeev S, Toka FN, Shiokawa K. Potential use of a canine whole blood culture system to evaluate the immune response to Leptospira. Comp Immunol Microbiol Infect Dis. 2020;73:101546. doi: 10.1016/j.cimid.2020.101546 32916553

[46] Inthasin N, Boonwong C, Matamnan S, Sueasuay J, Wongprompitak P, Tanttibhedhyangkul W, et al. Toll-like receptor 2-mediated induction of human beta-defensin 2 expression by Leptospira interrogans in human kidney cells. Asian Pacific J allergy Immunol. 2023 Dec;41(4):389–95. doi: 10.12932/AP-010420-0798 33068363

[47] Inthasin N, Wongprompitak P, Boonwong C, Ekpo P. Role of Toll-like receptor 2 in mediating the production of cytokines and human beta-defensins in oral mucosal epithelial cell response to Leptospiral infection. Regulation. 2018;10:12. 10.12932/AP-100518-0308 30118246

[48] Mercy CSA, Natarajaseenivasan K. hTLR2 interacting peptides of pathogenic leptospiral outer membrane proteins. Microb Pathog. 2021;155:104895. doi: 10.1016/j.micpath.2021.104895 33878396

[49] Varma VP, Bankala R, Kumar A, Gawai S, Faisal SM. Differential modulation of innate immune response by lipopolysaccharide of Leptospira. Open Biol. 2023 Nov;13(11):230101. doi: 10.1098/rsob.230101 37935355 PMC10645091

[50] Bernardi FDC, Ctenas B, da Silva LFF, Nicodemo AC, Saldiva PHN, Dolhnikoff M, et al. Immune receptors and adhesion molecules in human pulmonary leptospirosis. Hum Pathol. 2012;43(10):1601–10. doi: 10.1016/j.humpath.2011.11.017 22436623

[51] Lindow JC, Tsay AJ, Montgomery RR, Reis EAG, Wunder EA Jr, Araújo G, et al. Elevated activation of neutrophil toll-like receptors in patients with acute severe leptospirosis: an observational study. Am J Trop Med Hyg. 2019;101(3):585. doi: 10.4269/ajtmh.19-0160 31333152 PMC6726964

[52] Novak A, Pupo E, Van’t Veld E, Rutten VPMG, Broere F, Sloots A. Activation of Canine, Mouse and Human TLR2 and TLR4 by Inactivated Leptospira Vaccine Strains. Front Immunol. 2022;13:823058. 35386703 10.3389/fimmu.2022.823058 PMC8978998

[53] Guo Y, Fukuda T, Donai K, Kuroda K, Masuda M, Nakamura S, et al. Leptospiral lipopolysaccharide stimulates the expression of toll‐like receptor 2 and cytokines in pig fibroblasts. Anim Sci J. 2015;86(2):238–44. doi: 10.1111/asj.12254 25039909

[54] Guo Y, Fukuda T, Nakamura S, Bai L, Xu J, Kuroda K, et al. Interaction between Leptospiral lipopolysaccharide and Toll-like receptor 2 in pig fibroblast cell line, and inhibitory effect of antibody against Leptospiral lipopolysaccharide on interaction. Asian-Australasian J Anim Sci. 2015;28(2):273. doi: 10.5713/ajas.14.0440 25557825 PMC4283174

[55] Charo N, Scharrig E, Ferrer MF, Sanjuan N, Carrera Silva EA, Schattner M, et al. Leptospira species promote a pro‐inflammatory phenotype in human neutrophils. Cell Microbiol. 2019;21(2):e12990. doi: 10.1111/cmi.12990 30537301

[56] Akino Mercy CS, Suriya Muthukumaran N, Velusamy P, Bothammal P, Sumaiya K, Saranya P, et al. MicroRNAs Regulated by the LPS/TLR2 Immune Axis as Bona Fide Biomarkers for Diagnosis of Acute Leptospirosis. Msphere. 2020;5(4):e00409–20. doi: 10.1128/mSphere.00409-20 32669469 PMC7364213

[57] Freitas TMS, Dias JM, Guimarães LKP, Peixoto SV, Silva RHS da, Badr KR, et al. Genomic Association between SNP Markers and Diseases in the “Curraleiro Pé-Duro” Cattle. Genes (Basel). 2021;12(6):806. 34070451 10.3390/genes12060806 PMC8228838

[58] Lacroix-Lamandé S D’Andon MF, Michel E, Ratet G Philpott DJ Girardin SE, et al. Downregulation of the Na/K-ATPase pump by leptospiral glycolipoprotein activates the NLRP3 inflammasome. J Immunol. 2012;188(6):2805–14. doi: 10.4049/jimmunol.1101987 22323544

[59] Werts C. Interaction of Leptospira with the innate immune system. Spirochete Biol Post Genomic Era. 2017;163–87. 10.1007/82_2017_46 29038956

[60] Tapping RI, Akashi S, Miyake K, Godowski PJ, Tobias PS. Toll-Like Receptor 4, But Not Toll-Like Receptor 2, Is a Signaling Receptor for Escherichia and Salmonella Lipopolysaccharides. J Immunol. 2000;165(10):5780–7. doi: 10.4049/jimmunol.165.10.5780 11067937

[61] Que-Gewirth NLS, Ribeiro AA, Kalb SR, Cotter RJ, Bulach DM, Adler B, et al. A methylated phosphate group and four amide-linked acyl chains in Leptospira interrogans lipid A: the membrane anchor of an unusual lipopolysaccharide that activates TLR2. J Biol Chem. 2004;279(24):25420–9. 15044492 10.1074/jbc.M400598200 PMC2556802

[62] Cerqueira TB, Athanazio DA, Spichler AS, Seguro AC. Renal involvement in leptospirosis: new insights into pathophysiology and treatment. Brazilian J Infect Dis. 2008;12(3):248–52. doi: 10.1590/s1413-86702008000300016 18833411

[63] Goncalves-de-Albuquerque CF, Burth P, Silva AR, Younes-Ibrahim M, Castro-Faria-Neto HC, Castro-Faria M V. Leptospira and inflammation. Mediators Inflamm. 2012;2012. doi: 10.1155/2012/317950 23132959 PMC3485547

[64] Monahan AM, Callanan JJ, Nally JE. Host-pathogen interactions in the kidney during chronic leptospirosis. Vet Pathol. 2009;46(5):792–9. 19429975 10.1354/vp.08-VP-0265-N-REV

[65] Tian YC. Leptospirosis and Kidney Fibrosis. In: Leptospirosis and the Kidney. Karger Publishers; 2019. p. 57–64.

[66] Yang CW. Leptospirosis renal disease: understanding the initiation by Toll-like receptors. Kidney Int. 2007;72(8):918–25. doi: 10.1038/sj.ki.5002393 17687261

[67] Napetschnig J, Hao W. Molecular Basis of NF-kB Signaling. 2013;443–68. 10.1146/annurev-biophys-083012-130338 PMC3678348 23495970

[68] Senavirathna I, Rathish D, Agampodi S. Cytokine response in human leptospirosis with different clinical outcomes: a systematic review. BMC Infect Dis. 2020;20(1):1–8. 10.1186/s12879-020-04986-9 PMC7137275 32264832

[69] Kim NH, Sung JY, Choi YJ, Choi SJ, Ahn S, Ji E, et al. Toll-like receptor 2 downregulation and cytokine dysregulation predict mortality in patients with Staphylococcus aureus bacteremia. BMC Infect Dis. 2020;20(1):1–10. doi: 10.1186/s12879-020-05641-z 33256638 PMC7706030

[70] Koymans KJ, Goldmann O, Karlsson CAQ, Sital W, Thänert R, Bisschop A, et al. The TLR2 Antagonist Staphylococcal Superantigen-Like Protein 3 Acts as a Virulence Factor to Promote Bacterial Pathogenicity in vivo. J Innate Immun. 2017;9(6):561–73. doi: 10.1159/000479100 28858870

[71] Bardoel BW, Vos R, Bouman T, Aerts PC, Bestebroer J, Huizinga EG, et al. Evasion of Toll-like receptor 2 activation by staphylococcal superantigen-like protein 3. J Mol Med. 2012;90(10):1109–20. doi: 10.1007/s00109-012-0926-8 22714643

[72] Yokoyama R, Itoh S, Kamoshida G, Takii T, Fujii S, Tsuji T, et al. Staphylococcal superantigen-like protein 3 binds to the toll-like receptor 2 extracellular domain and inhibits cytokine production induced by Staphylococcus aureus, cell wall component, or lipopeptides in murine macrophages. Infect Immun. 2012;80(8):2816–25. doi: 10.1128/IAI.00399-12 22665377 PMC3434575

[73] Koymans KJ, Feitsma LJ, Bisschop A, Huizinga EG, Van Strijp JAG, De Haas CJC, et al. Molecular basis determining species specificity for TLR2 inhibition by staphylococcal superantigen-like protein 3 (SSL3) 06 Biological Sciences 0601 Biochemistry and Cell Biology. Vet Res [Internet]. 2018;49(1):1–15. Available from: 10.1186/s13567-018-0609-8 30486901 PMC6263051

[74] Friedman RC, how Farh KK, Burge CB, Bartel DP. Most mammalian mRNAs are conserved targets of microRNAs. 2009;92–105. doi: 10.1101/gr.082701.108 18955434 PMC2612969

[75] Benz F, Roy S, Trautwein C, Roderburg C, Luedde T. Circulating MicroRNAs as biomarkers for sepsis. Int J Mol Sci. 2016;17(1). doi: 10.3390/ijms17010078 26761003 PMC4730322

[76] Huang T, Yang J, Zhang J, Ke W, Zou F, Wan C, et al. MicroRNA-101-3p Downregulates TLR2 Expression, Leading to Reduction in Cytokine Production by Treponema pallidum–Stimulated Macrophages [Internet]. Vol. 140, Journal of Investigative Dermatology. Society for Investigative Dermatology; 2020. 1566-1575.e1 p. Available from: 10.1016/j.jid.2019.12.012 31930972

[77] Landais I, Pelton C, Streblow D, Defilippis V. Human Cytomegalovirus miR-UL112-3p Targets TLR2 and Modulates the TLR2 / IRAK1 / NF κ B Signaling Pathway. 2015;1–21. 10.1371/journal.ppat.1004881 PMC4425655 25955717

[78] Fouts DE, Matthias MA, Adhikarla H, Adler B, Amorim-Santos L, Berg DE, et al. What Makes a Bacterial Species Pathogenic?:Comparative Genomic Analysis of the Genus Leptospira. PLoS Negl Trop Dis. 2016;10(2):1–57. doi: 10.1371/journal.pntd.0004403 26890609 PMC4758666

