# Supplementary material for: Role of Toll-like receptor 2 during infection of Leptospira spp: A systematic review
Source: PLoS One. 2024 Dec 27;19(12):e0312466. doi: 10.1371/journal.pone.0312466 (PMC11676585; doi:10.1371/journal.pone.0312466)
Supplement: S1 Table — (DOCX) [file pone.0312466.s001.docx]

| Database | Search string |
| --- | --- |
| PubMed | **(((Leptospiraceae[MeSH Terms]) OR (Leptospira[Title/Abstract])) OR (((((((((((((Leptospirosis[MeSH Terms]) OR (Leptospiroses[Title/Abstract])) OR ("Leptospira Infection*"[Title/Abstract])) OR ("Stuttgart Disease"[Title/Abstract])) OR ("Mud Fever"[Title/Abstract])) OR ("Canicola Fever"[Title/Abstract])) OR ("Weil's Disease"[Title/Abstract])) OR ("Weils Disease"[Title/Abstract])) OR ("Spirochetal Jaundice"[Title/Abstract])) OR ("Icterohemorrhagic Leptospirosis"[Title/Abstract])) OR ("Leptospira Canicola Infection"[Title/Abstract])) OR ("Leptospirosis Canicola"[Title/Abstract])))) AND ((((((("Toll-like receptor 2"[MeSH Terms]) OR ("Toll Like Receptor 2"[Title/Abstract])) OR ("TLR2 Receptor"[Title/Abstract])) OR ("Toll-like receptor 2"[Title/Abstract])) OR ("Toll-Like Receptor 2"[Title/Abstract])) OR ("TLR-2"[Title/Abstract])) OR ("TLR2"[Title/Abstract]))** |
| Trip | (Toll-Like Receptor 2 OR Toll Like Receptor 2 OR TLR2 Receptor OR TLR2 OR TLR-2 OR Toll like receptor 2) AND (Leptospirosis OR Leptospiroses OR Leptospira Infection OR Leptospira Infections OR Stuttgart Disease OR Mud Fever OR Swineherd's Disease OR Swineherd's Diseases OR Canicola Fever OR Weil's Disease OR Weils Disease OR Spirochetal Jaundice OR Icterohemorrhagic Leptospirosis OR Leptospira Canicola Infection OR Leptospirosis Canicola OR Leptospiraceae OR Leptospira) |
| Scopus | TITLE-ABS-KEY ( "Toll-Like Receptor 2"  OR  "Toll Like Receptor 2"  OR  "TLR2 Receptor"  OR  tlr2  OR  tlr-2  OR  "Toll like receptor 2" )  AND  TITLE-ABS-KEY ( leptospir*  OR  "Mud Fever"  OR  "Swineherd's Disease"  OR  "Swineherd's Diseases"  OR  "Canicola Fever"  OR  "Weil's Disease"  OR  "Weils Disease" ) |
| Web of Science | TS=(“Toll-Like Receptor 2” OR “Toll Like Receptor 2” OR “TLR2 Receptor” OR “TLR2” OR “TLR-2”OR “Toll like receptor 2”) AND TS=(“Leptospirosis” OR “Leptospiroses” OR “Leptospira Infection*”OR “Stuttgart Disease” OR “Mud Fever” OR “Swineherd&#39;s Disease*” OR “Canicola Fever” OR “Weil’s Disease” OR “Weils Disease” OR “Spirochetal Jaundice” OR “Icterohemorrhagic Leptospirosis” OR “Leptospira Canicola Infection” OR “Leptospirosis Canicola” OR “Leptospiraceae” OR “Leptospira”) |

S1 Table. Search strings
