# Supplementary material for: Role of Toll-like receptor 2 during infection of Leptospira spp: A systematic review
Source: PLoS One. 2024 Dec 27;19(12):e0312466. doi: 10.1371/journal.pone.0312466 (PMC11676585; doi:10.1371/journal.pone.0312466)
Supplement: S2 Table — (DOCX) [file pone.0312466.s002.docx]

| **Study ID** | **Country of origin** | **In vitro/In vivo/Human** | **Incubation period** |
| --- | --- | --- | --- |
| Goris,2011 | The Netherlands | In vitro | 6 hours |
| Werts,2001 | USA | In vitro+ In vivo | In vitro 6 hours  In vivo 90 minutes |
| Yang,2006 | Taiwan | In vitro | 48 hrs |
| Nahori,2005 | France | In vivo + In vitro | 24 hrs |
| Chassin,2009 | France | In vivo | 3days |
| Mercy,2020 | India | Human, In vivo, and In vitro | 0,4,7,14,21 days |
| Viriyakosol,2006 | USA | In vivo and In vitro | 16 hrs |
| Chang,2016 | Taiwan | In vivo(zebra fish larvae) | 5 days |
| Charo,2019 | Argentina | Human neutrophils(Ex vivo, in vitro) | Flow-4hr, ELISA-3-18hr,RT qPCR- |
| Chou,2018 | Taiwan | In vivo | 7 day |
| Faisal,2016 | India | In vitro(Mouse macro,RAW264.7) | 24 hrs |
|  |  | In vitro and In vivo |  |
| (Guo,2015)-1 | Japan | In vitro(pig fibroblast cell line) | 6,12,24 hrs |
| (Guo,2015)-2 | Japan | In vitro(pig fibroblast cell line) | 3-72hrs |
| (Guo,2016) | China | In vitro(bovine cells) | 6 hrs |
| Hsu,2021 | Taiwan | In vitro | 2 hrs, 4hrs |
| (Hung,2006)-1 | Taiwan | In vitro(mice) | 24 hrs |
| (Hung,2006)-2 | Taiwan | In vitro(mice PTCs,HEK 293) | 24 hrs |
| Inthasin,2018 | Thailand | Human oral cells(Ex vivo, in vitro) | 4 hours |
| Liu,2021 | China | In vivo | 4 days |
| Raffray,2019 | France | Human | NR |
| Raffray,2016 | France | Human | NR |
| Rajeev,2020 | USA | Canine blood(Ex vivo) | ELISA(1,2,6,24h) PCR array (18h) |
| Santecchia,2019 | France | In vivo | 24 hrs |
| Tian,2011 | Taiwan | In vitro | 24-48 h |
| Wang,2012 | China | Human, In vitro, In vivo(mice) | 24 h |
| Bernadi,2012 | Brazil | In vitro | 6h-24h |
| Zhang,2010 | China | In vitro(rat kidney cells) | 48h |
| Zhang,2016 | China | In vitro, In vivo | 6,12,24hrs,2day |
| Zhang,2020 | China | In vivo(Hamsters) | 2 days |
| Yijie,2016 | China | In vitro(Bovine) | 6 hours |
| Lindow,2019 | Brazil | Human | 1-7 days |
| Bonhomme,2024 | France | In vitro (Mice) | 24 hours |
| Inthasin,2023 | Thailand | In vitro (Human Kidney Epithelium cells) | 6 hours |
| Novak,2022 | Netherlands | In vitro (Canine,human,mouse TLR2 expressing HEK Blue reporter cells) | 24 hours |
| Varma,2023 | India | In vitro(Murine macrophages, HEK cell line,THP1  Cell line ) | 24 hours |
