# Supplementary material for: Role of Toll-like receptor 2 during infection of Leptospira spp: A systematic review
Source: PLoS One. 2024 Dec 27;19(12):e0312466. doi: 10.1371/journal.pone.0312466 (PMC11676585; doi:10.1371/journal.pone.0312466)
Supplement: S5 Table — (DOCX) [file pone.0312466.s005.docx]

| Study ID | Quality assessment of case-control studies (NIH developed) | | | | | | | | | | | | |
| --- | --- | --- | --- | --- | --- | --- | --- | --- | --- | --- | --- | --- | --- |
|  | Q1 | Q2 | Q3 | Q4 | Q5 | Q6 | Q7 | Q8 | Q9 | Q10 | Q11 | Q12 | Rating |
| *Akino,2020 | *Y | Y | *NR | Y | Y | Y | *NR | NR | Y | Y | Y | NR | 08/12 |
| Raffray,2016 | Y | Y | NR | Y | Y | Y | NR | NR | Y | Y | Y | NR | 08/12 |
| Raffray,2019 | Y | Y | NR | Y | Y | Y | NR | NR | Y | Y | Y | NR | 08/12 |
| *Wang,2012 | Y | Y | NR | Y | Y | Y | NR | NR | Y | Y | Y | NR | 08/12 |
| Lindow,2019 | Y | Y | NR | Y | Y | Y | NR | NR | Y | Y | Y | NR | 08/12 |
| *CD, cannot determine; *Y,Yes; *N,No; *NA, not applicable; *NR, not reported *Akino,2020 *Wang,2012; Combined studies; Human+Invitro+Invivo. Q1.Was the research question or objective in this paper clearly stated and appropriate? Q2.Was the study population clearly specified and defined? Q3.Did the authors include a sample size justification? Q4.Were controls selected or recruited from the same or similar population that gave rise to the cases (including the same timeframe)? Q5.Were the definitions, inclusion and exclusion criteria, algorithms or processes used to identify or select cases and controls valid, reliable, and implemented consistently across all study participants? Q6.Were the cases clearly defined and differentiated from controls? Q7. If less than 100 percent of eligible cases and/or controls were selected for the study, were the cases and/or controls randomly selected from those eligible? Q8.Was there use of concurrent controls? Q9.Were the investigators able to confirm that the exposure/risk occurred prior to the development of the condition or event that defined a participant as a case? Q10.Were the measures of exposure/risk clearly defined, valid, reliable, and implemented consistently (including the same time period) across all study participants? Q11.Were the assessors of exposure/risk blinded to the case or control status of participants? Q12.Were key potential confounding variables measured and adjusted statistically in the analyses? If matching was used, did the investigators account for matching during study analysis?  Rating;(1-4 Poor) (5-8 Fair) (9-12 Good) | | | | | | | | | | | | | |

S5 Table. ROB assessment

Quality assessment of case-control studies (NIH developed)
