# Supplementary material for: Role of Toll-like receptor 2 during infection of Leptospira spp: A systematic review
Source: PLoS One. 2024 Dec 27;19(12):e0312466. doi: 10.1371/journal.pone.0312466 (PMC11676585; doi:10.1371/journal.pone.0312466)
Supplement: S6 Table — (DOCX) [file pone.0312466.s006.docx]

S6 Table. ROB assessment

SYRCLE’s risk of bias tool (Animal studies)

| ID | Selection Bias | | | Performance Bias | | Detection Bias | | Attrition  Bias | Reporting  Bias | Other |  |
| --- | --- | --- | --- | --- | --- | --- | --- | --- | --- | --- | --- |
|  | Q1 | Q2 | Q3 | Q4 | Q5 | Q6 | Q7 | Q8 | Q9 | Q10 | Rating |
| Chassin,2009 | UC | Yes | UC | UC | UC | UC | UC | Yes | Yes | Yes | 04/10 |
| Chang,2016 | UC | Yes | UC | UC | UC | UC | UC | Yes | Yes | Yes | 04/10 |
| Chou,2018 | UC | Yes | UC | UC | UC | UC | UC | Yes | Yes | Yes | 04/10 |
| Zhange,2020 | UC | Yes | UC | UC | UC | UC | UC | Yes | Yes | Yes | 04/10 |
| Liu,2021 | UC | Yes | UC | UC | UC | UC | UC | Yes | Yes | Yes | 04/10 |
| Santecchia,2019 | UC | Yes | UC | UC | UC | UC | UC | Yes | Yes | Yes | 04/10 |
| Wang,2012 | UC | Yes | UC | UC | UC | UC | UC | Yes | Yes | Yes | 04/10 |
| Akino,2020 | UC | Yes | UC | UC | UC | UC | UC | Yes | Yes | Yes | 04/10 |
| Wertz,2001 | UC | Yes | UC | UC | UC | UC | UC | Yes | Yes | Yes | 04/10 |
| Viriyakosol,2006 | UC | Yes | UC | UC | UC | UC | UC | Yes | Yes | Yes | 04/10 |
| Nahori,2005 | UC | Yes | UC | UC | UC | UC | UC | Yes | Yes | Yes | 04/10 |
| Zhang,2016 | UC | Yes | UC | UC | UC | UC | UC | Yes | Yes | Yes | 04/10 |
| Q1.Was the allocation sequence adequately generated and applied? Q2.Were the groups similar at baseline or were they adjusted for confounders in the analysis? Q3. Was the allocation adequately concealed? Q4. Were the animals randomly housed during the experiment?  Q5. Were the caregivers and/or investigators blinded from knowledge which intervention each animal received during the experiment? Q6. Were animals selected at random for outcome assessment? Q7. Was the outcome assessor blinded? Q8. Were incomplete outcome data adequately addressed? Q9. Are reports of the study free of selective outcome reporting? Q10. Was the study apparently free of other problems that could result in high risk of bias? Rating;(1-3 Poor)(4-6 Fair)(7-10 Good) | | | | | | | | | | | |
