# Supplementary material for: Role of Toll-like receptor 2 during infection of Leptospira spp: A systematic review
Source: PLoS One. 2024 Dec 27;19(12):e0312466. doi: 10.1371/journal.pone.0312466 (PMC11676585; doi:10.1371/journal.pone.0312466)
Supplement: S7 Table — (DOCX) [file pone.0312466.s007.docx]

S7 Table. ROB assessment

OHAT extended Quality Assessment for *In-vitro* and *ex-vivo* studies

| ID | Selection Bias | | | Confounding  bias | Performance  Bias | | Attrition  Bias | Detection Bias | | Selective  Reporting | Other |  |
| --- | --- | --- | --- | --- | --- | --- | --- | --- | --- | --- | --- | --- |
|  | Q1 | Q2 | Q3 | Q4 | Q5 | Q6 | Q7 | Q8 | Q9 | Q10 | Q11 | Rating |
| Goris,2011 | DL | DL | NA | NA | DL | NR | DL | DL | DL | DL | DL | 08/09 |
| Yang,2006 | DL | DL | NA | NA | DL | NR | DL | DL | DL | DL | DL | 08/09 |
| Faisal,2016 | DL | DL | NA | NA | DL | NR | DL | DL | DL | DL | DL | 08/09 |
| (Guo,2015)-1 | DL | DL | NA | NA | DL | NR | DL | DL | DL | DL | DL | 08/09 |
| (Guo,2015)-2 | DL | DL | NA | NA | DL | NR | DL | DL | DL | DL | DL | 08/09 |
| Guo,2016 | DL | DL | NA | NA | DL | NR | DL | DL | DL | DL | DL | 08/09 |
| Hsu,2021 | DL | DL | NA | NA | DL | NR | DL | DL | DL | DL | DL | 08/09 |
| (Hung,2006)-1 | DL | DL | NA | NA | DL | NR | DL | DL | DL | DL | DL | 08/09 |
| (Hung,2006)-2 | DL | DL | NA | NA | DL | NR | DL | DL | DL | DL | DL | 08/09 |
| Tian,2011 | DL | DL | NA | NA | DL | NR | DL | DL | DL | DL | DL | 08/09 |
| Bernadi,2012 | DL | DL | NA | NA | DL | NR | DL | DL | DL | DL | DL | 08/09 |
| Zhang,2010 | DL | DL | NA | NA | DL | NR | DL | DL | DL | DL | DL | 08/09 |
| Yijie,2016 | DL | DL | NA | NA | DL | NR | DL | DL | DL | DL | DL | 08/09 |
| Wang,2012 | DL | DL | NA | NA | DL | NR | DL | DL | DL | DL | DL | 08/09 |
| Akino,2020 | DL | DL | NA | NA | DL | NR | DL | DL | DL | DL | DL | 08/09 |
| Wertz,2001 | DL | DL | NA | NA | DL | NR | DL | DL | DL | DL | DL | 08/09 |
| Viriyakosol,2006 | DL | DL | NA | NA | DL | NR | DL | DL | DL | DL | DL | 08/09 |
| Nahori,2005 | DL | DL | NA | NA | DL | NR | DL | DL | DL | DL | DL | 08/09 |
| Zhang,2016 | DL | DL | NA | NA | DL | NR | DL | DL | DL | DL | DL | 08/09 |
| Rajeev,2020 | DL | DL | NA | NA | DL | NR | DL | DL | DL | DL | DL | 08/09 |
| Inthasin,2018 | DL | DL | NA | NA | DL | NR | DL | DL | DL | DL | DL | 08/09 |
| Charo,2019 | DL | DL | NA | NA | DL | NR | DL | DL | DL | DL | DL | 08/09 |
| Santecchia,2019 | DL | DL | NA | NA | DL | NR | DL | DL | DL | DL | DL | 08/09 |
| Bonhomme,2024 | DL | DL | NA | NA | DL | NR | DL | DL | DL | DL | DL | 08/09 |
| Inthasin,2023 | DL | DL | NA | NA | DL | NR | DL | DL | DL | DL | DL | 08/09 |
| Novak,2022 | DL | DL | NA | NA | DL | NR | DL | DL | DL | DL | DL | 08/09 |
| Varma,2023 | DL | DL | NA | NA | DL | NR | DL | DL | DL | DL | DL | 08/09 |
| DL-Definitely low, PL-Probably low, PH-Probably High, DH-Definitely High Q1. Was administered dose or exposure level adequately randomized? Q2. Was allocation to study groups adequately concealed? Q3. Did selection of study participants result in the appropriate comparison groups? Q4. Did study design or analysis account for important confounding and Modifying variables? Q5. Were experimental conditions identical across study groups? Q6. Were research personnel blinded to the study group during the study?  Q7. Were outcome data complete without attrition or exclusion from analysis? Q8. Can we be confident in the exposure characterization? Q9. Can we be confident in the outcome assessment (including blinding of assessors)? Q10. Were all measured outcomes reported? Q11. Were there no other potential threats to internal validity?  Rating;(1-3 Poor) (4-6 Fair) (7-9 Good) | | | | | | | | | | | | |
